# The draft genome of *Primula veris* yields insights into the molecular basis of heterostyly

**DOI:** 10.1186/s13059-014-0567-z

**Published:** 2015-01-24

**Authors:** Michael D Nowak, Giancarlo Russo, Ralph Schlapbach, Cuong Nguyen Huu, Michael Lenhard, Elena Conti

**Affiliations:** Institute of Systematic Botany, University of Zürich, Zollikerstrasse 107, 8008 Zürich, Switzerland; Natural History Museum, University of Oslo, P.O. Box 1172, Blindern NO-0318, Oslo, Norway; Functional Genomics Center Zürich, University of Zürich and ETH Zürich, Winterthurerstrasse 190, 8057 Zürich, Switzerland; Institut für Biochemie und Biologie, Universität Potsdam, Karl-Liebknecht-Strasse 24-25, Haus 26, 14476 Potsdam, Germany

## Abstract

**Background:**

The flowering plant *Primula veris* is a common spring blooming perennial that is widely cultivated throughout Europe. This species is an established model system in the study of the genetics, evolution, and ecology of heterostylous floral polymorphisms. Despite the long history of research focused on this and related species, the continued development of this system has been restricted due the absence of genomic and transcriptomic resources.

**Results:**

We present here a *de novo* draft genome assembly of *P. veris* covering 301.8 Mb, or approximately 63% of the estimated 479.22 Mb genome, with an N50 contig size of 9.5 Kb, an N50 scaffold size of 164 Kb, and containing an estimated 19,507 genes. The results of a RADseq bulk segregant analysis allow for the confident identification of four genome scaffolds that are linked to the *P. veris S*-locus. RNAseq data from both *P. veris* and the closely related species *P. vulgaris* allow for the characterization of 113 candidate heterostyly genes that show significant floral morph-specific differential expression. One candidate gene of particular interest is a duplicated *GLOBOSA* homolog that may be unique to *Primula* (*PveGLO2*), and is completely silenced in L-morph flowers.

**Conclusions:**

The *P. veris* genome represents the first genome assembled from a heterostylous species, and thus provides an immensely important resource for future studies focused on the evolution and genetic dissection of heterostyly. As the first genome assembled from the Primulaceae, the *P. veris* genome will also facilitate the expanded application of phylogenomic methods in this diverse family and the eudicots as a whole.

**Electronic supplementary material:**

The online version of this article (doi:10.1186/s13059-014-0567-z) contains supplementary material, which is available to authorized users.

## Background

With over 350,000 described species, angiosperms currently represent the dominant, most diverse group of plants on earth [1]. Their success has been frequently linked with the evolution of a complex structure, the flower, which typically includes both male and female sexual organs - often inconspicuous - inside whorls of attractive, asexual organs. This evolutionary innovation opened up a new landscape of opportunities for elaborate interactions with animals, mostly insects, which can transfer male gametes (that is, pollen grains) between flowers of different plants more efficiently than abiotic vectors (for example, wind; [2,3]). Most flowers are hermaphroditic, theoretically enabling fertilization within the same individual (selfing), a breeding system that can lead to detrimental evolutionary consequences [4,5]. Different strategies have thus evolved in flowering plants to avoid selfing and promote outcrossing, and one of the most effective mechanisms is heterostyly.

Extensively investigated in primroses (*Primula* L., Primulaceae) by Darwin [6], heterostyly refers to a floral polymorphism whereby individuals in a population produce dissimilar types of flowers (two in some taxa, three in others) with male and female sexual organs in different, but spatially matching positions [7]. For example, in *Primula*, plants produce two types of flowers (that is, distyly): either long-styled flowers with anthers attached midway along the floral tube (‘L-morph’, or ‘pin’), or short-styled flowers with anthers attached at the top of the floral tube (‘S-morph’, or ‘thrum’). Male and female sexual organs of L-morph and S-morph flowers, respectively, are thus placed in complementary positions, a condition known as reciprocal herkogamy. Heterostylous species frequently employ a genetic mechanism for rejecting pollen of the same plant and pollen from the same floral morph known as heteromorphic self-incompatibility [8]. The combination of reciprocal herkogamy and self-incompatibility enforces outcrossing [9,10], thus avoiding the potentially negative effects of inbreeding. The adaptive value of heterostyly may thus explain why this system has evolved repeatedly in the angiosperms, and is presently known to occur in at least 28 different families [11].

The best-studied group of heterostylous plants is arguably *Primula*, comprising 400 to 500 species, of which approximately 90% are distylous and 10% homostylous, the latter producing self-compatible flowers of only one type. Primroses occur primarily in temperate, alpine, and arctic habitats of the northern hemisphere, with the main center of species diversity in the Sino-Himalayan region and secondary centers in the major, circumboreal mountain chains [12]. Owing to its fascinating reproductive system, attractive flowers, and common occurrence in the historical centers of Western science, the genus has been intensively examined from comparative perspectives for at least 150 years. Numerous studies are thus available on phylogenetic relationships within the genus (for example, [13,14]), biogeography, ecology, and variation of ploidy levels (for example, [15-19]), floral and reproductive biology (for example, [6,9,10,20,21]), genetics of heterostyly (for example, [22-25]), evolution of breeding systems (13, for example, [26-28]), and conservation ecology and genetics (for example, [29,30]). Furthermore, primroses also represent one of the most popular garden plants in Europe, Japan, and North America, valued as spring-blooming perennials and rock-garden alpines. Primroses are prominent in the horticultural trade, and business associated with the sale of plants, organization of exhibitions, and collecting expeditions is estimated at 50 million USD annually (J. Richards, personal communication).

Within the genus, *Primula* section *Primula* has received the most scientific attention, starting with Darwin’s [6] seminal work on the floral morphology and reproductive biology of *Primula veris* (cowslip), *Primula vulgaris* (primrose), and *Primula elatior* (oxlip). The section includes six distylous and one homostylous species, all diploids with a base chromosome number of 11 (that is, 2n = 2x = 22). Typical elements of the spring flora in many parts of Eurasia, these three species are the most widespread in the section, ranging from Western Europe to central and even far-Eastern Asia [31]. Their abundance and easy accessibility in Europe may partially explain why they have been investigated so intensively. A broad range of studies have been performed on reproductive isolation and hybridization among these three species ([9,10], for example, [32-41]), pollination biology, ecology and conservation (for example, [42-46]), floral morphology, self-incompatibility, and the genetics of distyly (for example, [47-52]).

The genus, and in particular *Primula* sect. *Primula*, have thus emerged as a model for a broad spectrum of evolutionary, ecological, and conservation studies, and as the paradigm for exploring the genetic control of distyly [7,53]. Classic genetic studies on primroses, based on manual crosses and segregation analyses, established that distyly is governed by a single Mendelian locus (the *S*-locus), with long-styled and short-styled plants being homozygous (*ss*) and heterozygous (*Ss*), respectively, and the homozygous dominant genotype (*SS*) being lethal [54-57]. A minimum of three genes are thought to be tightly linked at the *S*-locus (sometimes called the S-supergene), controlling the length of the style (*Gg*: short; *gg*: long), the position of the anthers (*Aa*: high; *aa*: low), the size of pollen grains (*Pp*: large; *pp*: small) and other ancillary traits ([23], for example, the length of the stigmatic papillae [24,58,59]). Large populations often harbor individuals with intermediate phenotypes suggestive of rare recombination events at the *S*-locus. Lewis and Jones [23] used the frequency of such recombinants to infer the linkage order of the three loci as *GPA* in *Primula* subgenus *Auriculastrum*, but Kurian and Richards [24] found that the gene order was more likely *GAP* in *Primula* section *Primula*. Beyond the relative order of the three linked loci, little is known regarding the absolute size of the *Primula S*-locus, as well as the number and function of specific genes that these loci represent [8]. To date, just three genes have been identified as potentially linked to the *Primula S*-locus, and none of these have been proposed as functionally integral to the differential development of L- and S-morph flowers. In 2007, Li *et al.* [60] employed fluorescent differential display to identify, clone, and sequence L- and S-morph alleles of two genes that they named *PvSLL1* and *PvSLL2*, which represent an unknown plasma membrane protein, and a gene with sequence similarity to *Arabidopsis* flower-timing genes *Col9* and *Col10*, respectively. While searching for the genes responsible for the *S*-locus-linked floral homeotic mutant *sepaloid* in *Primula vulgaris*, Li *et al.* [60] identified *PvGLO*, a homolog to the B-function MADS-box gene *GLOBOSA*. While their results suggested that *PvGLO* was not likely responsible for the mutant *sepaloid* phenotype, the gene appeared to carry alleles exhibiting morph-specific segregation, and it was thus assumed to be linked to the *Primula S*-locus.

Given that the *Primula S*-locus is largely shielded from recombination, there are certain well-established theoretical predictions that can be made regarding the genome sequence harboring the *S*-locus. The classic model of Muller’s Ratchet predicts that reduced recombination will lead to the fixation of a large number of linked sites on both L-morph and S-morph *S*-locus haplotypes [61]. This implies that both coding and non-coding sequence residing within the boundaries of the *S*-locus should exhibit elevated heterozygosity in S-morph (*Ss*) plants relative to L-morph plants (*ss*).

Despite the plethora of evolutionary, ecological, morphological, and genetic studies on *Primula* and its longstanding value as a prime biological study system, we still lack the genomic resources that would allow us to perform detailed analyses of speciation processes, identify areas of the genome that are more porous to introgression, characterize the genetic basis of adaptation to alpine/arctic habitats, exploit the genes that control traits of special horticultural value, and finally elucidate the enduring mystery of the molecular basis of distyly. In turn, the molecular characterization of the *S*-locus would open up new avenues of research into the evolutionary pathways leading to heterostyly [27,62,63] and the repeated evolution and loss of this trait in angiosperms [14,28,64]. Here, we present the first draft genome assembly of a heterostylous species, focusing on *Primula veris*, because this species has long been a favorite target of scientific studies (as reviewed above) and represents a well-defined evolutionary lineage [31]. This species is diploid with a chromosome number of 2n = 22 and estimated DNA content of 479.22 Mb [65]. Our aim in assembling the *P. veris* genome is primarily to develop genomic resources for this species and the entire genus *Primula*, to identify genome scaffolds that segregate with the *P. veris S*-locus, and to use these newly created resources to examine genes previously identified as linked to the *Primula S*-locus and characterize novel candidate distyly genes. Our genome and transcriptome assemblies enable the continued development of *P. veris* as a model system to study the genetic components of the *S*-locus and the evolution and maintenance of reproductive barriers within *Primula* sect. *Primula*.

## Results and discussion

### Genome and transcriptome assemblies

We generated a large amount of sequence data from a diverse suite of sequencing libraries to assemble the draft *P. veris* genome. A full account of our sequencing efforts is shown in Table 1. Using these data, we employed a two-step strategy for *de novo* genome assembly in order to fully leverage the long-read data generated by PacBio RS. Our first assembly was performed using only short-read (that is, 100 to 250 bp) sequences generated from standard paired-end and 3 to 9 Kb mate-pair libraries on Illumina HiSeq, MiSeq, and Ion Proton platforms. This assembly was based on a total of 54.5 Gb of raw data and resulted in a total of 48,812 contigs that were grouped into 9,002 unique scaffolds (Additional file 1: Table S1). The total contig length is 232.2 Mb and the total scaffold length, including gaps, is 301.8 Mb, which represent 49% and 63%, respectively, of the estimated 479.22 Mb *P. veris* genome [65]. The N50 contig size is 9.5 Kb, and the N50 scaffold size including gaps is 164 Kb, with a median gap size of 960 bp. The largest and smallest scaffolds are 2.14 Mb and 888 bp, respectively.

**Table 1:** **Summary of sequence data used in the** ***de novo*** **assembly of the draft** ***P. veris*** **genome**

**Library type**	**Platform**	**Reads**	**Bases**	**Coverage** ^**a**^	**Insert size** ^**a**^ **(bp)**	**Read length** ^**a**^ **(bp)**
Standard paired-end	Illumina HiSeq	222,499,994	22,249,999,400	50	180	100
Mate pair (small)	Illumina HiSeq	59,854,090	5,985,409,000	15	3,298	100
	Illumina MiSeq	109,369,174	8,340,785,860	19	3,348	76
	Ion Proton	35,503,706	1,584,426,676	4	3,412	44
Total		204,726,970	15,910,621,536	38		
Mate pair (medium)	Illumina HiSeq	37,705,200	3,770,520,000	9	6,048	100
	Illumina MiSeq	43,084,090	3,590,695,375	8	6,084	83
Total		80,789,290	7,361,215,375	17		
Mate pair (large)	Illumina HiSeq	55,721,478	5,572,147,800	13	8,900	100
	Illumina MiSeq	42,153,704	3,425,486,254	8	8,938	81
Total		97,875,182	8,997,634,054	21		
Total (short-read data)			32,269,470,965	116		
Long read fragment 10 kb	PacBio RS	2,357,643	3,437,918,089	7	NA	3,658^b^

^a^Average values reported.

^b^See also Additional file 2: Figure S1.

The transcriptome sequencing of *P. veris* and *P. vulgaris* yielded more than 200 million paired reads, which were distributed quite evenly among the six RNAseq libraries that we multiplexed and sequenced in parallel on a single Illumina flow cell (Additional file 1: Table S2). Our *de novo P. veris* transcriptome assembly contains 25,409 putatively unique transcripts, and the *P. vulgaris* transcriptome assembly contains 24,318 unique transcripts. We also performed *de novo* transcriptome assemblies of the related species *P. obconica*, *P. wilsonii*, and *P. poissonii* using data available on the Genbank Sequence Read Archive (SRA). Our *de novo* transcriptome assembly of *P. obconica* was found to contain 22,752 transcripts, but the transcriptome assemblies of *P. poissonii* and *P. wilsonii* were considerably smaller, with 11,905 and 12,927 unique transcripts, respectively. This reduced transcript pool likely represents the fact that the publicly available Genbank SRA data for these species contained far fewer raw sequence reads [66] than those we produced for *P. veris* and *P. vulgaris*, and than the reads available in the SRA for *P. obconica*.

To improve our *P. veris* genome assembly, we generated 3.4 Gb of PacBio RS sequence data with an average read length of 3,658 and an average base quality of 0.834 (Additional file 2: Figure S1). These data, which contribute an additional 8× coverage for the *P. veris* genome, were integrated into our assembly through the implementation of the PbJelly software tool [67]. The PBJelly algorithm essentially anchors the long PacBio reads to the existing contigs and scaffolds and extends them partially spanning the contiguous gaps. When PacBio sequences are long enough to be anchored to both sides of a gap, then the gap is fully closed. Adding the PacBio RS data to the assembly resulted in a total of 21.15% of the gaps in the previous assembly being entirely closed and 38.4% of the ambiguous positions in the gaps being filled. This translated into a significant improvement of both the compactness and completeness of our *de novo P. veris* genome. At 269.7 Mb, the new total contig length increased by 19.4%; the new total scaffold length (including gaps) reached 310.07 Mb, a 2.7% increase over the draft assembly based on the Illumina and Ion Proton data alone. In terms of genome completeness, these contig-length numbers represent 56.3% and 64.7%, respectively, of the *P. veris* genome. The updated draft is also more compact: the number of contigs was reduced, by 16.88%, to 40,569 contigs, grouped into 8,764 unique scaffolds (2.65% fewer scaffolds, see Additional file 1: Table S1). At 13.3 Kb, the N50 contig size grew by 40%, whereas the N50 scaffold size including gaps reached 165.9 Kb, with a median gap size of 664 bp. The largest and smallest contigs are 233,407 bp and 19 bp, respectively. The sizes of the largest and smallest scaffolds remained 2.14 Mb and 888 bp, respectively; this is not surprising, since gap-closing tends to bridge scaffolds of low to medium size. The assembly scaffolds on average contain 29 ambiguities per 10 kb. The GC content of the scaffolds, excluding gaps, is 33%, which is similar to the genome sequences of tomato (34%; [68]) and kiwifruit (35.2%; [69]).

Although the success of a draft genome assembly is strongly dependent on the genetic complexity of the specific organism and its genome size, we find the overall quality of the draft *P. veris* assembly, based on the aforementioned measures, to be consistent with the majority of *de novo* genome assembly efforts reported in the last few years. Unsurprisingly, genome assemblies generated from highly homozygous samples, such as the PN40024 grapevine line [70], or those employing a larger number of *ad hoc* mate pair libraries (for example, domesticated apple *Malus* × *domestica* [71]; kiwifruit *Actinidia chinensis* [69]; mulberry *Morus notabilis* [72]) resulted in higher quality drafts exhibiting a three- to four-fold decrease in the number of scaffolds and a two- to four-fold increase in the corresponding N50, relative to our assembly. However, when compared to assemblies in which similar resources were used, our assembly is quite compact. For example, our *P. veris* assembly is between two and 10 times more compact than the draft genomes of *Cannabis sativa* [73] and European pear (*Pyrus communis* ‘Bartlett’ [74]), which contain 136,290 and 142,083 scaffolds, respectively, and scaffold N50s smaller than 90 Kb. These genome projects employed two mate pair libraries of sizes similar to our study, but it is important to point out that their minimum contig size was less than 500 bp, while our assembly is based on a minimum contig size of 1,000 bp. Our assembly is one of the first to specifically employ PacBio sequence data to fill scaffold gaps in a heterozygous plant genome, and this aspect, together with the use of a third, larger mate pair library, may be responsible for the improved compactness (that is, fewer contigs) of our assembly.

### Genome annotation and quality assessment

Of the 8,764 scaffolds that make up our genome assembly, 2,495 (28.5%) were found to contain annotated genes. Our final annotation of the *P. veris* genome contained a total of 19,507 predicted genes, less than the number of genes found in the relatively well-annotated genomes of *Arabidopsis thaliana* (27,029 genes; [75]), sacred lotus (*Nelumbo nucifera*, 26,685 genes; [76]), and mulberry (29,338 genes; [72]). Furthermore, we find about 7,000 fewer genes predicted in the genome annotation compared with the *de novo P. veris* transcriptome assembly (25,409 predicted genes; see above), but most likely the real number of genes is somewhere in between those two estimates, and a combination of factors might have contributed to underestimating the predictions in the genome annotation and inflating the number of genes predicted in the *de novo* transcriptome assembly. First, transcripts predicted by the *de novo* transcriptome assembly are based on only one source of evidence, that is, the RNA-seq data, and represent only one source of evidence in the Maker2 genome annotation process. For a transcript to be reported by Maker2, at least two sources of evidence are required, hence those transcripts that are not strongly supported by a second source of evidence, such as *ab initio* gene prediction algorithms or protein homology are not reported in the final genome annotation. Additionally, a *de novo* transcriptome assembly alone is often not able to completely discriminate between multiple isoforms of a specific gene, which may slightly inflate the estimated number of genes based on these data alone. The predicted gene content of *P. veris* is significantly reduced compared to kiwifruit (39,040 genes [69]) and European pear (43,413 genes [74]), probably owing to the relatively recent whole genome duplications in the latter two species. The Maker2 pipeline identified a total of 279,271 repetitive elements representing approximately 7% of the *P. veris* draft genome assembly (Additional file 1: Table S3).

To assess the completeness of the final genome assembly, we searched for the presence of 248 conserved core eukaryotic genes using the CEGMA software package (CEGs; [77]). We found confident hits to 198 (79.84%) full length (that is, >70% alignment) CEG proteins and 234 partial (94.35%) CEG proteins, where partial matches are defined by the CEGMA default pre-computed minimum alignment score for each CEG [77]. These results suggest an exceptional level of completeness for this first draft genome sequence of a heterozygous, non-model plant. Annotated scaffolds contained a median number of four genes, and the largest scaffold (Contig0; 2.15 Mb) was found to contain 352 predicted genes, and generally, longer scaffolds tend to contain more genes. The quality of the gene prediction and annotation was evaluated in a number of ways. Searching the predicted gene set with the InterProScan tool, we found that 15,659 (85.6%) of predicted proteins contain PFAM domains. One commonly applied quality metric of genome annotations is the cumulative annotation edit distance (AED), which represents the agreement between predicted gene models and external evidence. A gene model with an AED score of zero indicates complete agreement between the predicted gene model and, for example, a transcript from the *de novo P. veris* transcriptome assembly, which we incorporate as external evidence in the Maker2 pipeline [78]. As the *ab initio* gene predictors are trained through iterative Maker2 runs, the cumulative fraction of predicted gene models with low AED scores increases, thus indicating more agreement between gene models and external evidence (Additional file 3: Figure S2). In our final annotation, approximately 80% of gene models have an AED score of 0.2 or less, indicative of a high degree of agreement between predicted gene models and external evidence.

### Comparative transcriptome analyses

**Figure 1:**
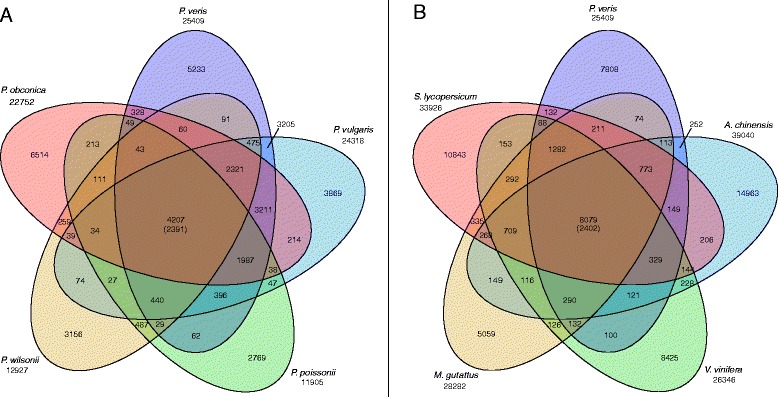
**Venn diagrams of orthologous gene clusters.** The number of putatively unique transcripts in each transcriptome is shown below the taxon name. The value in parentheses represents the number of orthologous genes that are most likely to be single-copy in all of the five transcriptomes sampled. **(A)** Orthologous groups identified in comparative transcriptome analysis of five species of *Primula*. **(B)** Orthologous groups identified in comparative transcriptome analysis of five Euasterid species with draft genome assemblies. See also Additional files 4 and 5.

We used the OrthoMCL pipeline [79,80] to identify sets of putatively orthologous loci at two phylogenetic scales: (1) among transcriptomes of five species of *Primula*; and (2) among *P. veris* and four Euasteridae species with published genome assemblies (Figure 1). Our comparative *Primula* transcriptome analyses confidently identified a total of 4,207 orthologs among the transcriptomes of *P. veris*, *P. vulgaris*, *P. obconica, P. wilsonii*, and *P. poissonii*, with 2,391 of these likely representing single-copy genes in all of the species (Figure 1A; see Additional file 4). The most recent phylogenetic hypotheses for the genus *Primula* suggest that *P. veris*, *P. vulgaris* [31] and *P. poissonii*, *P. wilsonii*, respectively, belong to two different sister clades, [66], with *P. obconica* included in a third clade basal to the other two clades [13,14]. Our results are consistent with these hypothesized phylogenetic relationships, for we find more shared orthologs between *P. vulgaris* and *P. veris* than in all other pairwise comparisons (3,205), and a similar number of putatively orthologous genes limited to *P. veris*, *P. vulgaris*, and *P. obconica* (3,211). However, we do not see a similar number of shared orthologs unique to the sister species *P. poissonii* and *P. wilsonii* (487), but this result might be explained by the fact that these transcriptomes were assembled with far less sequence data [66] than our *de novo* transcriptome assemblies, and thus they are likely to represent a smaller proportion of the expressed genes. Our comparative analyses of euasterid transcriptomes identified a total of 8,079 putatively orthologous genes, and of these approximately 2,402 are likely to be single copy in all examined species (Figure 1B; see Additional file 5). These orthologous gene sets provide a framework for the development of future phylogenomic studies aimed at resolving species-level relationships within the genus *Primula*, or deeper phylogenetic relationships within the Euasteridae.

### Gene expression differences between floral morphs and functional analysis of candidate genes

To search for genes that might underlie the phenotypic differences between the floral morphs in *Primula*, we compared gene expression between flowers of L- and S-morph plants in both *P. vulgaris* and *P. veris*. Given that we sampled RNA from floral bud tissues 3 to 5 days prior to anthesis, our results are primarily relevant to the later stage of floral development. At this stage, cell growth in the style and in the corolla tube below the point of stamen attachment differs between L- and S-morph flowers. Differential cell growth is thought to be primarily responsible for differences in style length and the relative position of the anthers in the middle or the top of the corolla tube, respectively [48]. We adopted a Benjamini-Hochberg false discovery rate (FDR) at 5% level to correct for multiple testing. At an FDR-adjusted *P* value threshold of 0.05, we found 620 and 677 genes in *P. veris* and *P. vulgaris*, respectively, that exhibited significant differential expression between morphs. To reduce the number of false positives in this gene set, we retained only the 113 genes that exhibit morph-specific differential expression in both *P. veris* and *P. vulgaris* floral buds (Additional file 1: Table S4). Of these 113 genes, the majority (that is, 73 genes) showed increased expression in S-morph versus L-morph floral buds. Because the distylous phenotype is limited to floral tissues, it is likely that genes involved in determining the floral morph would not be differentially expressed in L- and S-morph leaf tissues. We find that, of these 113 genes showing floral morph-specific differential expression, 69 are not differentially expressed in *P. veris* L- and S-morph leaves (Additional file 1: Table S4).

**Figure 2:**
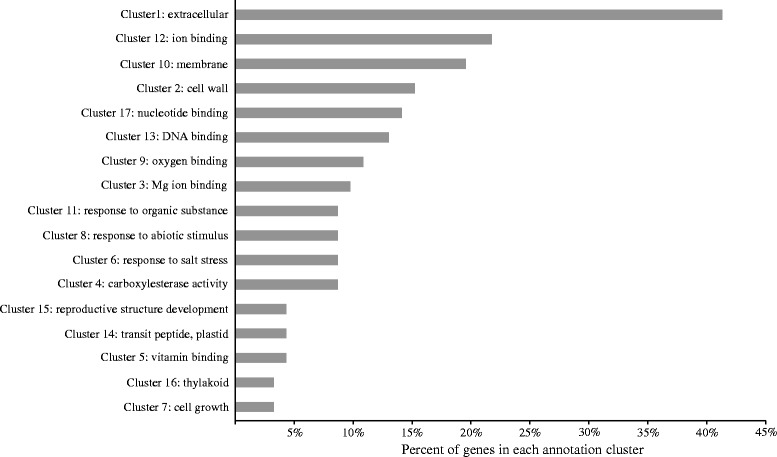
**Distribution of genes differentially expressed in L- and S-morph plants assigned to functional annotation clusters.** Annotation clusters are labeled with a single GO term that is representative of all of the terms defining cluster membership. See Additional file 1: Table S5 for a complete listing of GO terms associated with each annotation cluster.

We examined the candidate set of 113 differentially expressed genes to determine the types of functional gene classes represented. Functional annotations for 92 of the 113 genes were clustered using the DAVID bioinformatics resources, while the remaining 21 genes contained no conserved protein domains and are not readily assignable to functional classes. Annotation clusters represent groups of functionally similar GO terms associated with the gene list, thus providing a more direct biological interpretation of related terms for large gene lists [81]. As seen in Figure 2, GO terms attributed to the 92 genes showing morph-specific differential expression can be grouped into 17 annotation clusters. More than 40% of the candidate genes involved in differential floral morph expression contain GO terms attributable to annotation cluster 1, which is characterized by the terms ‘extracellular’, ‘secreted’, and ‘hydrolase’, among others (Additional file 1: Table S5). These terms could be related to a wide array of biological processes, but it is plausible that some of the genes might control mechanisms of heteromorphic self-incompatibility typical of distylous *Primula* species. Self- and intra-morph pollen tube inhibition in *Primula* is thought to result from biochemical interactions between the pollen grain or pollen tube and tissues of the stigma and/or style [82,83]. Such biochemical interactions are likely to occur in the extracellular matrix or at the stigmatic surface, thus requiring the active secretion of molecules involved in the heteromorphic self-incompatibility mechanism [52,84-87].

The remaining 16 annotation clusters contain GO terms associated with 25% to approximately 3% of the candidate genes; among these are a few clusters that stand out as being potentially relevant to the phenotypic differences associated with the two floral morphs. Specifically, annotation clusters 2 and 7 are characterized by GO terms associated with cell-wall organization and cell growth, respectively. These clusters are particularly interesting in the context of morph-specific development, because many of the phenotypic differences that define the L- and S-morph flowers involve differential cell proliferation and elongation in the corolla tube (the *A* gene) and the style (the *G* gene, [12,48,88]). Annotation cluster 15 is characterized by the terms ‘post-embryonic development’, ‘reproductive structure development’, and ‘reproductive developmental process’ (Additional file 1: Table S5). Floral development genes frequently act as transcription factors regulating the expression of a diverse suite of genes that influence the development of specific floral structures [89]. It is thus possible that the genes associated with the GO terms of annotation cluster 15 could be coordinating the developmental changes associated with differential cell elongation observed in corolla tubes and styles of L- and S-morph plants.

### GLO1 & GLO2: Duplicated GLOBOSA homologs in Primula

**Figure 3:**
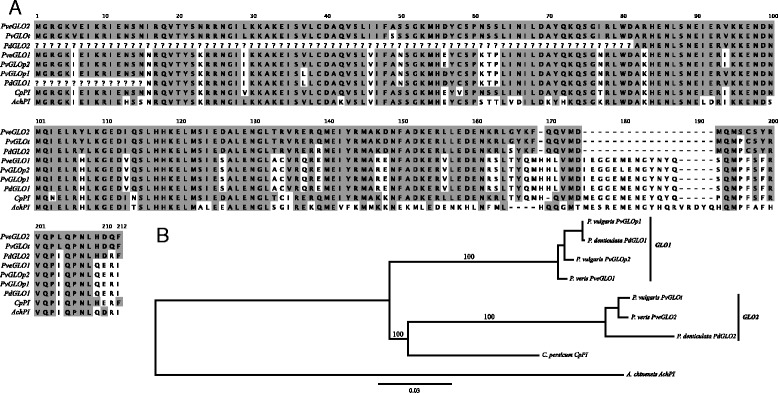
**Sequence alignment and tree showing the relationship of** ***GLOBOSA*** **/** ***PISTILLATA*** **genes in Primulaceae. (A)** Amino acid positions that are shaded are identical to *PveGLO2*. **(B)** The tree was rooted with the *PI* gene sequence from the kiwifruit draft genome (*A. chinensis*, [69]). Branch support values were calculated based on 1,000 neighbor-joining bootstrap replicates. Our labelling of *GLO1* and *GLO2* follows the convention established by Viaene *et al.* [91]. See text for details regarding gene names.

One of the most intriguing results of our differential expression analysis in *P. veris* and *P. vulgaris* is the fact that BG8816696, the gene with the most significant morph-specific differential expression (as measured by FDR-adjusted *P* value; Additional file 1: Table S4), is strikingly similar to the B-function MADS-box gene *GLOBOSA* (*E*-value = 0), which is a transcription factor integral in the normal development of petals and stamens in *Antirrhinum majus* [90]. Blast searches reveal that this gene had been previously sequenced from *P. vulgaris* by Li *et al.* [60] and from *P. denticulata* by Viaene *et al.* [91]. While Li *et al.* [60] considered this gene to be single copy, Viaene *et al.* [91] realized that there were actually two *GLOBOSA* homologs in *P. denticulata*. When we map the *GLOBOSA* homologs from *P. vulgaris* (*PvGLO*^*P1*^, *PvGLO*^*P2*^, and *PvGLO*^*T*^) and *P. denticulata* (*PdGLO1* and *PdGLO2*) to our genome assembly, we find unequivocal support for two distinct *GLOBOSA* genes in *Primula*. Specifically, we find that Li *et al.*’s [60] two putative L-morph alleles (named *PvGLO*^*P1*^ and *PvGLO*^*P2*^ by the authors) and *PdGLO1* [91] map to an entirely different scaffold than the putative S-morph allele (*PvGLO*^*T*^ [60]) and *PdGLO2* [91]. We hereby refer to these two genes as *PveGLO1* (BG8816827), which is homologous to *PdGLO1* and the two alleles *PvGLO*^*P1*^ and *PvGLO*^*P2*^ [60], and *PveGLO2* (BG8816696), which is homologous to *PdGLO2* and *PvGLO*^*T*^ (Figure 3). Viaene *et al.* [91] failed to find evidence for two *GLOBOSA* homologs in a monomorphic species of Primulaceae (*Cyclamen persicum*), and thus it is possible that the duplicated *GLOBOSA* could be specific to the genus *Primula*. By comparing the mRNA sequences of *PvGLO*^*T*^ [60], *PdGLO2* [91], and *PveGLO2* assembled from S-morph RNAseq data with the scaffold sequence carrying *PveGLO2* (Contig1404), we find that the last exon and 3’ UTR appear inverted in the genome assembly. This inversion in the scaffold sequence is likely the product of an assembly error, because the transcriptome assembly contains a complete *PveGLO2* gene and there is a large (approximately 10 Kb) assembly gap within the last intron of the *PveGLO2* gene model. *PveGLO2* is completely silenced in L-morph flowers of *P. veris* and *P. vulgaris*, which - in light of Li *et al.*’s [60] finding of linkage between *PveGLO2* (*PveGLO*^*T*^ in their terminology) and the S-morph haplotype of the *S*-locus in *P. vulgaris* - could reflect the absence of this gene from the L-morph haplotype. Thus, while we currently do not have evidence for *PveGLO2* being linked to the *S*-locus in *P. veris* (see below), this duplicated B-function MADS-box gene is an attractive candidate for future studies of distylous flower development in *Primula*.

### Bulk Segregant RAD-Seq identifies SNPs linked to the *P. veris S-*locus

To enable testing of the above hypotheses about properties of *S-*locus linked sequences, we aimed to experimentally identify corresponding scaffolds. To this end, we searched for sequence polymorphisms tightly linked to the *S*-locus in *P. veris*, using a RAD-seq bulk segregant analysis on pooled L-morph and S-morph DNA. Any polymorphisms that are closely linked to the *S-*locus should be largely homozygous in the L-morph pool, but heterozygous in the S-morph pool. Using a stringent set of criteria (coverage in both pools >60×, allele frequency in L-morph pool 1.0, allele frequency in S-morph pool 0.3-0.7), this analysis identified 24 SNPs as potentially tightly linked to the *S* locus (see Additional file 6).

**Figure 4:**
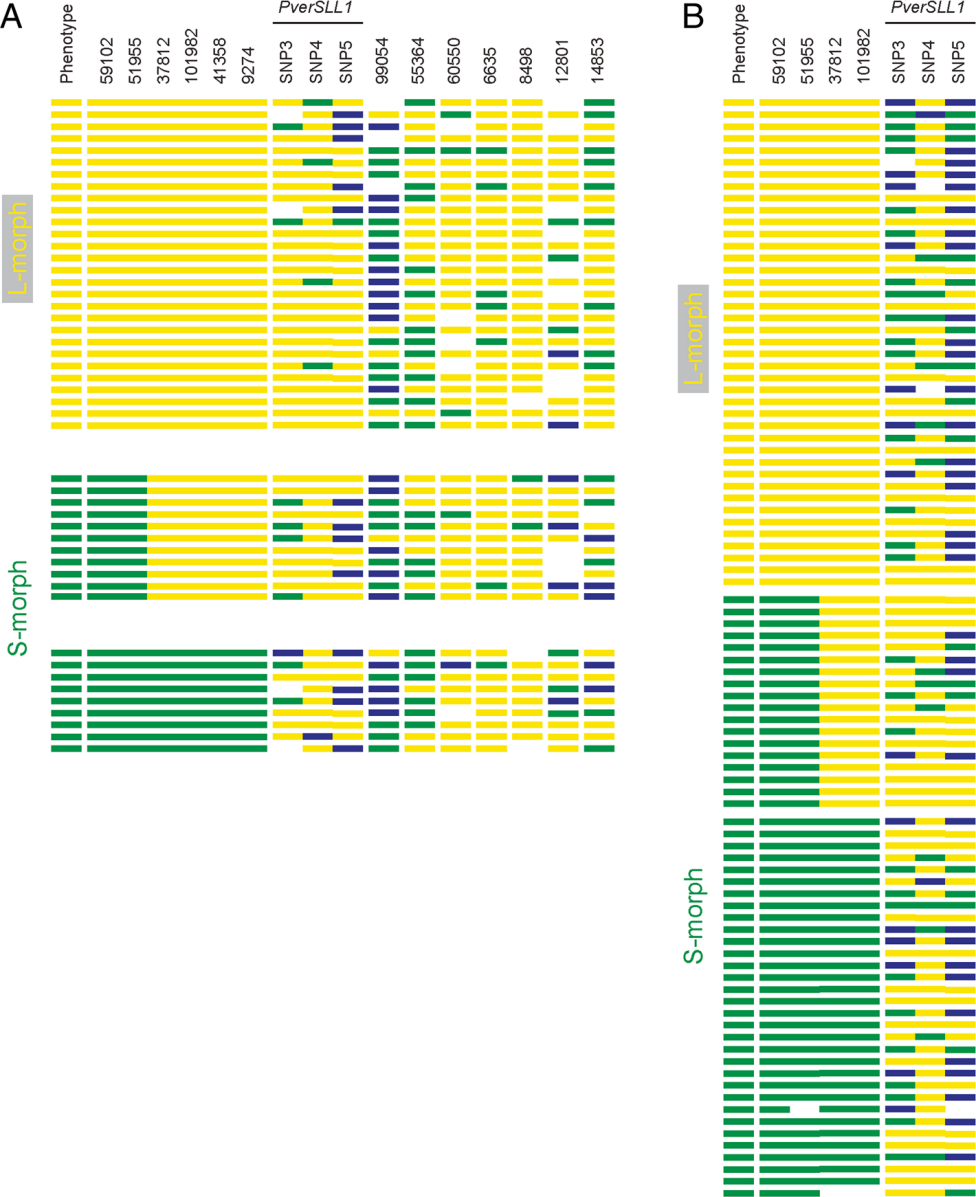
**Identification of** ***S*** **-locus linked SNPs.** Graphical representation of genotypes of **(A)** 48 *P. veris* plants genotyped for 13 PCR-based markers derived from the bulk segregant RAD sequencing analysis and three SNPs in *SLL1*, and **(B)** 91 *P. veris* plants genotyped for four PCR-based SNP markers and three SNPs in the *SLL1* gene. Phenotype: yellow = L-morph, green = S-morph. Genotypes: yellow = homozygous for allele 1, green = heterozygous, blue = homozygous for allele 2.

To enable high-throughput genotyping, the SNPs identified above were converted to PCR-based markers, either cleaved-amplified polymorphic sequences (CAPS) or derived CAPS (dCAPS). These markers were initially tested on pooled L- or S-morph DNA. Out of the 24 markers, 13 allowed for robust amplification and detected the predicted polymorphisms between L- and S-morph pools. These markers were next used to genotype 48 individuals from a natural *P. veris* population in Potsdam, Germany, and based on these data, six markers were found to be linked to the *S-*locus, while the remaining seven showed no clear correlation between marker genotype and *S*-locus phenotype (that is, floral morph; Figure 4). Inspection of the genotypes in Figure 4A indicates that markers 9274, 37812, 41358, 51955, 59102, and 101982 are completely linked to the *S-*locus when considering only the L-morph plants. The genotypes of S-morph plants suggest that in the population there are in fact two common chromosomes harboring the dominant *S*-allele (S-morph). The first is found in phenotypically S-morph plants that are heterozygous for all six markers in question and thus carries S-morph specific alleles at these markers; this chromosome will be termed the ‘original S-morph chromosome’ below. The other common chromosome is found in phenotypically S-morph plants homozygous for the L-morph allele at markers 9274, 37812, 41358, and 101982. Hence, a recombination event appears to have combined the dominant *S* allele with the recessive *s* (L-morph) alleles at these four markers, and the resulting ‘recombinant S-morph chromosome’ appears to have spread in the local population. No recombination was found among the four markers 9274, 37812, 41358, and 101982. Thus, a tentative, relative marker order deduced from these 48 samples is: (*S-*locus/51955/59102) - (37812/101982/9274/41358), where the markers/loci in brackets cannot be separated from each other. From these six markers we chose four (51955, 59102, 37812, 101982) and genotyped 91 additional individuals (Figure 4B). No further recombinant genotypes beyond the ones found in the first 48 plants were detected, thus confirming the deduced order of loci. By blasting the six loci to the *P. veris* genome assembly, we find that markers 37812 and 101982 are both located on Contig437, a result consistent with their complete linkage in the mapping results. Notably, we also find that markers 9274 and 59102 are separated by approximately 70 Kb on Contig273. Our mapping results suggest that the recombination event that may have given rise to the ‘recombinant S-morph chromosome’ could have occurred between 9274 and 59102 on Contig273, and this region can thus be considered a functional boundary of the *S*-locus. We also note that the absence of any detectable recombination between four markers (37812, 101982, 9274, and 41358) located on three different scaffolds of considerable size outside of the functional *S-*locus indicates that the suppression of recombination extends over large distances beyond the *S-*locus (see Table 2).

**Table 2:** **Genome scaffolds putatively linked to the** ***P. veris S*** **-locus**

**Scaffold ID**	**Length**	**Genes**	**RE**	**DE genes**	**L-morph RNA**	**S-morph RNA**	**S-/L-morph SNP ratio**	**Comments**
Contig1892	36,436	1	39	0	5	5	0	*PvSLL1* (Li *et al.* [49])
Contig927	105,749	7	104	1^a^	18	20	1.1	*PvSLL2* (Li *et al.* [49])
Contig2830	45,782	4	38	0	10	11	1.1	*PveGLO1* (*PvGLO* ^*P1/P2*^; Li *et al.* [60])
Contig1404	48,939	1	31	1	0	0	0	*PveGLO2* (*PvGLO* ^*T*^; Li *et al.* [60])
Contig437	106,280	6	52	0	8	40	5	RAD loci 37812 and 101982
Contig478	326,606	21	379	2	29	39	1.3	RAD locus 41358
Contig578	132,036	14	119	1^b^	16	96	6	RAD locus 51955
Contig273	185,956	11	132	1^c^	62	98	1.5	RAD loci 9274 and 59102

^a^Differentially expressed only in *P. veris* floral tissues.

^b^Differentially expressed only in *P. vulgaris* floral tissues.

^c^Differentially expressed only in *P. veris* leaf tissues.

SNPs identified by mapping RNAseq reads from L- and S-morph flowers to the genome assembly are presented.

DE Genes = the number of genes on the scaffold that show significant morph-specific differential expression; RE = number of repetitive elements predicted.

To compare the resolution afforded by our mapping in the natural *P. veris* population to previous reports, we isolated the *P. veris* locus orthologous to the previously described *PvSLL1* locus from *P. vulgaris* [49]. Comparing the sequences of the *P. veris* and the *P. vulgaris SLL1* locus indicated that one of the internal repeats found in *P. vulgaris* is replaced by an unrelated sequence in *P. veris* (Additional file 7: Figure S3). Sequencing this locus from pools of L- and S-morph individuals identified three polymorphic SNPs that were converted to PCR-based genotyping markers. Testing the 139 samples above for these three SNPs indicated no association of any of the SNPs with the *S-*locus genotype, nor an association of any of the resulting seven haplotypes present in the sample with the *S-*locus genotype (Figure 4). Although there was still significant linkage disequilibrium between the three SNPs (Additional file 8: Figure S4; Χ^2^-test; *P* <0.0001), we identified a substantial proportion of recombinant genotypes, even though the three SNPs are all found within less than 200 bp. This suggests that the studied population captures a very large number of historical recombination events resulting in an absence of LD between *PvSLL1* and the *S* locus. Conversely, it indicates that the very tightly linked markers identified above are likely to reside within or immediately next to the *S-*locus.

### Genome-wide estimates of heterozygosity and polymorphism

Heterozygosity and polymorphism were explored in the *P. veris* genome by resequencing a single *P. veris* individual from a Latvian population and mapping these raw sequence reads to our genome assembly. We found a total of 1,184,748 SNPs (0.51%) composed of 982,590 heterozygous sites and 202,158 homozygous sites compared to the reference assembly, and a total of 96,113 indels were identified in the Latvian *P. veris* individual (see Additional file 9).

Theory predicts that the *S-*locus will be largely shielded from recombination [92], and thus *S*-linked genome scaffolds are expected to show elevated rates of heterozygosity in S-morph plants, which are heterozygous at the *S*-locus (Ss), compared to L-morph plants, which are homozygous at the *S*-locus (ss). We evaluated this prediction by mapping RNAseq reads from L- and S-morph plants of *P. veris* to our genome assembly to compare the number of SNPs predicted in coding sequences of genome scaffolds that are putatively linked to the *S*-locus. We found a total of 45,018 SNPs when mapping the L-morph RNA to the genome assembly, and 51,191 SNPs when mapping the S-morph RNA to the genome assembly, and the average S-morph/L-morph SNP ratio for all genome scaffolds is 2.54 (variance = 23.78). Table 2 shows the results of this analysis for specific genome scaffolds carrying genes that have been previously identified as linked to the *S*-locus in *P. vulgaris*: *PvSLL1*, *PvSLL2* [49], *PveGLO1*, and *PveGLO2* [60]. Additionally, we include the genome scaffolds carrying the six loci that are linked to the *S*-locus based on our RAD-seq bulk segregant analysis and fine mapping experiments (see above): RAD loci 41,358, 37,812/101,982, 51,955, and 9,274/59,102. Our results show that, on average, there are more SNPs mapped from S-morph RNA reads than L-morph RNA reads on putatively *S*-linked scaffolds (Table 2). By utilizing RNAseq reads rather than whole genome sequence data, our analysis is limited to polymorphisms evident in coding regions alone. Additionally, if certain genes experience high degrees of allele-specific expression, we will fail to observe polymorphisms solely due to an absence of mapped reads. It is important to note here that Contig1404, which carries just one gene (*PveGLO2*), contains no polymorphism when mapping either L- or S-morph RNAseq reads: this is the expected result if *PveGLO2* were *S*-linked and only expressed in the *S*-locus of the S-morph. The expectation of increased heterozygosity is fundamentally based on the assumption that individuals with an L-morph phenotype will also be highly homozygous at the *S*-locus. But if recombination is suppressed between L-morph alleles as well, then one might actually expect that linked polymorphisms would accumulate between different L-morph haplotypes, thus leading to elevated heterozygosity at the *S*-locus in L-morph as well as S-morph plants. Addressing this question is beyond the scope of the current study, but future analyses aimed at more precisely characterizing genomic patterns of heterozygosity in and around the *S*-locus will benefit greatly from our draft genome assembly of *P. veris*.

## Conclusions

Our *P. veris* genome assembly exemplifies the power of high-throughput DNA sequencing technologies and establishes a benchmark for the rapid *de novo* assembly of a highly heterozygous, non-model plant with a moderately sized genome (that is, 479.22 Mb). We believe that the primary strength of our sequencing strategy lies in the diversity of sequence libraries and sequencing platforms we have employed. Our study suggests great promise in the application of PacBio long-read data for the improvement of *de novo* genome assemblies, and it is possible that, with the direct incorporation of the raw PacBio sequences in the assembly process, even more information could be extracted from long reads. Currently such integration is strongly limited by the *a priori* correction of raw PacBio reads, which significantly reduces the quantity of usable data resulting from a PacBio run (G. Russo, personal observation). In the case of large eukaryotic genomes, this translates into the need for a largely unfeasible sequencing throughput.

Using our *de novo* genome assembly coupled with RNAseq data from flower buds, we have identified 113 genes that show significant morph-specific differential expression in both *P. veris* and *P. vulgaris*. Functional analysis of the list of candidate genes has revealed clusters of GO terms related to extracellular processes, cell growth and organization, and development of reproductive structures. Furthermore, our genome assembly shows that the B-function MADS box gene *GLOBOSA* has been duplicated in *Primula*, and we find that one of these copies (*PveGLO2*) is silenced in L-morph flower buds, but we still do not know if *PveGLO2* is linked to the *S*-locus, and thus a suitable candidate gene for morph-specific floral development. Future work toward characterizing this candidate gene could involve resequencing both L- and S-morph plants to evaluate the presence of this gene in both morphs and identify morph-specific SNPs that can be tested for *S*-locus linkage in a mapping population.

Employing a bulk segregant analysis followed by high-resolution mapping, we identify six loci on four genome scaffolds that are tightly linked to the *S*-locus in *P. veris*. When examining these *S*-linked genome scaffolds as well as genome scaffolds carrying genes previously identified as linked to the *S*-locus, we find elevated heterozygosity in S-morph versus L-morph coding sequences, consistent with theoretical predictions. Future work toward defining the recombinational limits and genetic composition of the *S*-locus would benefit greatly from the construction of a linkage map using genetic markers anchored within genome scaffolds. Beyond characterizing the *Primula S*-locus, our *de novo* genome sequence will prove to be a valuable resource for marker design and the analysis of population genomic and phylogenomic data. The genomic resources presented here thus represent a significant leap forward in the development of *P. veris* and *P. vulgaris* as models in the study of distyly, climatic adaptation, and speciation genetics.

## Materials and methods

### DNA isolation, library preparation, and sequencing

Genomic DNA was extracted from one L-morph and one S-morph individual of *P. veris* individuals from a cultivated line maintained by the Vogt Stauden nursery [93]. Genomic DNA was extracted from young leaf tissue using two different protocols. The CelLytic PN Isolation/Extraction Kit (Sigma-Aldrich, Buchs, Switzerland) was used to partially enrich nuclear DNA by following the manufacturer’s protocol for ‘Crude Preparation of Nuclei’, and the resulting product was further purified using a standard CTAB DNA extraction protocol [94]. This genomic DNA is herein referred to as ‘partially enriched genomic DNA’. A second sample of ‘total genomic DNA’ was directly extracted from young leaves following the standard CTAB DNA extraction protocol [94]. For use in estimating the genomic levels of polymorphism (see below), total genomic DNA was extracted from a *P. veris* individual from a population in the Mazsalaca Municipality in Latvia (57.91494°N, 24.98437°E). The floral morph of this plant was not noted when the sample was collected, and thus we do not know if this was a L- or S-morph individual. All DNA extractions were quantified using the Broad Range DNA Kit on a Qubit v.2.0 spectrophotometer (Life Technologies, Zug, Switzerland), and the presence of high molecular weight DNA was confirmed by 1% agarose-gel electrophoresis stained with 1% ethidium bromide.

Genomic DNA was prepared for sequencing by generating three different types of libraries. An overview of the library types employed for the assembly and their summary statistics are shown in Table 1. The small fragment library was prepared using the Illumina Paired-End DNA protocol. The average fragment size was 180 nucleotides and the fragments were sequenced on an Illumina HiSeq 2000 in paired-end mode. The length of 100 bases for both the forward and reverse reads ensured a small overlap across the read pairs, a feature recommended for *de novo* assembly. Mate-pair libraries were produced by circularizing DNA fragments of different lengths using a biotinylated internal adapter. The circular fragments were then sheared and the biotinylated segments were detected and amplified for sequencing. Illumina Nextera long mate-pair protocol and Life Technologies mate-pair protocol, which are based on the above-mentioned approach, were both applied and the resulting libraries were sequenced on Illumina HiSeq2000 and MiSeq instruments and on Life Technologies Ion Proton System, respectively. Libraries prepared from fragments of three different sizes (3 Kb, 6 Kb, and 9 Kb) were selected for sequencing on the Illumina technologies, whereas only libraries generated from 3 Kb fragments were sequenced on the Ion Proton. Finally, long fragment DNA templates were produced following the Pacific Biosciences (PacBio) 10 kb library protocol and sequenced on 25 SMRT cells of the PacBio RS II.

### Genome assembly

Fragments sequenced in paired-end mode on the Illumina HiSeq were stripped of the sequencing adapters without further preprocessing, due to the high quality of the sequencing run. Sequences produced from the mate-pair libraries had both external and internal adapters removed. Mate-pairs resulting from templates sequenced on platforms generating reads of variable length (that is, MiSeq and IonProton) were retained only if both the forward and the reverse reads contained at least 30 nucleotides after adapter removal. Long fragments sequenced on the Pacific Bioscience RSII had the SMRTbell adapters removed and filtered according to a minimum read length of 50 nucleotides and a minimum read quality of 75%.

The final draft of the *P.veris* genome was obtained using a two-step procedure.

*De novo* assembly was performed using ALLPATHS-LG v48579 [95], with specific settings for a diploid organism (that is, by setting PLOIDY = 2) and provided with a genome size estimate of 445 Mb [65]. Data from the genomic templates sequenced on the Pacific Biosciences (PacBio) RS II were not used at this stage, as ALLPATHS-LG relies on paired reads information (either paired-end or mate pairs) and discourages the use of long fragment reads when assembling large eukaryotic organisms. Long fragment reads were only successively incorporated to perform gap-closing and further scaffolding of the first assembly draft using PBJelly v14.1.15 [67]. The PacBio read-alignment software ‘blasr’ was provided with the following settings: *minMatch* = 8, *sdpTupleSize* = 8, *minPctIdentity* =  75, *bestn* = 1, *nCandidates* = 10, *maxScore* = 500, and *noSplitSubreads* was activated.

### RNA isolation, library preparation, and sequencing

With the aim of sequencing a diverse transcript pool, total RNA was isolated separately from floral and leaf tissues for each plant sampled in our study. Entire, mature floral buds, 3 to 5 days prior to anthesis were used for RNA extraction from floral tissues. Leaf tissues were sampled approximately 4 to 6 days prior to complete leaf blade expansion. RNA was isolated from a *P. veris* L-morph plant (Vogt 026-P) and from a *P. veris* S-morph plant (VE8-T). For comparative analyses, we also extracted floral bud RNA from *P. vulgaris* L-morph (VU2-P) and S-morph (VU-T37) plants 3 to 5 days prior to anthesis. Total RNA was extracted using the RNAqueous Kit (Life Technologies, Zug, Switzerland) following the manufacturer’s standard protocol. Extracted RNA was quantified using a Qubit v2 fluorometer RNA assay kit (Life Technologies) and diluted to 40 ng/μL for subsequent library construction. For all six samples, the standard unstranded Illumina TruSeq protocol was used, which includes a step to pull down polyadenylated transcripts from the cDNA pool. Libraries were then multiplexed using a set of six Illumina barcodes and sequenced on one lane of an Illumina HiSeq 2000 instrument in paired-end mode.

### Transcriptome assembly and comparative analysis

Transcriptome assemblies were generated for each species using either the raw Illumina sequence data from *P. veris* and *P. vulgaris* RNAseq runs or data downloaded from Genbank for *P. obconica*, *P. wilsonii*, and *P. poissonii* [66]. Adaptor sequences were removed from the raw sequencing reads, and reads with a quality score below 15 were removed. *De novo* transcriptome assembly was performed using the Trinity software suite v.2013-8-14 [96,97] using default parameters. The likely coding DNA sequences (CDS) and corresponding proteins within the *de novo* transcriptome assembly were estimated with Transdecoder [98]. Redundant transcripts potentially representing sequencing errors or genetic polymorphisms were clustered with the cd-hit software [99].

Protein sequences from the cd-hit results of the *P. veris*, *P. vulgaris*, *P. obconica*, *P. wilsonii*, and *P. poissonii* transcriptomes were used to identify clusters of putatively orthologous genes using OrthoMCL v.2.0.9 [79,80]. Pairwise sequence similarities between all protein sequences were calculated with blastp using an e-value cutoff of 1e-05. Using these results, protein clusters that are roughly equivalent to orthologous groups were estimated using the Markov clustering algorithm employed in OrthoMCL with the default inflation value (−I) of 1.5. A second OrthoMCL analysis was performed using identical parameters in order to identify putative orthologs with *P. veris* among a broader sample of sequenced and annotated Euasteridae genomes, including tomato (*Solanum lycopersicum*; [68]), grape (*Vitis vinifera*; [70]), kiwifruit (*Actinidia chinensis*; [69]), and monkeyflower (*Mimulus guttatus*; Mimulus Genome Project, DoE Joint Genome Institute).

### Gene prediction and annotation

Genome annotation was performed using the Maker2 pipeline [78]. Augustus v2.7 [100] and SNAP (Release 2013-11-29; [101]) were used as *ab initio* gene predictors. The Trinity-assembled *P. veris* transcripts (see above) were used as transcript evidence. Protein sequences from the *Arabidopsis thaliana* genome (version TAIR10) were used as homology-based evidence. Repetitive genomic elements were identified and masked from annotation with RepeatMasker [102] using the Repbase database [103]. The Maker2 annotation was first run using the *de novo* transcriptome directly to infer gene predictions (that is, est2genome = 1), and training files for the *ab initio* gene predictors Augustus and SNAP were generated based on these results. The annotation pipeline was then run iteratively two additional times using the assembled *P. veris* transcriptome as evidence (that is, est2genome = 0) and providing new training files with each run. At this point, the protein-homology set was broadened to include all Uniprot proteins in the Embryophyta and a final annotation was performed by also providing Maker2 with the transcript-annotation file (in .gff format) obtained from the previous runs (that is, ‘pass-through’ aided annotation). InterProScan v.5 was used to identify PFAM domains [104] in the predicted *P. veris* protein sequences using default parameters [105]. The predicted *P. veris* protein sequences were searched against a set of 248 highly conserved core eukaryotic genes using the CEGMA pipeline [77].

### Estimating heterozygosity and polymorphism

To estimate genome-wide patterns of heterozygosity and polymorphism, a single *P. veris* individual from near the northern range limit of the species in Latvia (see above) was resequenced. A total of 158.8 million paired-end reads were generated on one-third of a single lane of an Illumina HiSeq instrument, yielding about 16 Gb of data. The resulting reads were mapped to the newly constructed *P. veris* reference assembly using Bowtie2 [106] with paired-end settings and *--local-sensitive* parameters. Insertion/deletions and SNPs were called using a combination of SAMtools v0.1.19 and Bcftools v0.1.19 [107] with default settings. Polymorphisms reported as purely diploid with a minimum coverage of 30×, a maximum coverage of 300×, and a minimum quality score of 50 were retained.

Genomic regions within and tightly linked to the S-locus are expected to be more heterozygous in S-morph than in L-morph plants [92]. To identify genome scaffolds with elevated heterozygosity in coding regions of S-morph versus L-morph plants, we mapped the raw RNAseq reads from L-morph and S-morph individuals back to the *P. veris* genome assembly. The positions of SNPs and indels in the *P. veris* draft genome assembly are provided in Additional files 9 and 10.

### Gene expression differences between floral morphs

In order to perform differential expression analysis, the final assembly was used as reference genome and the genomic features annotated by Maker2 acted as gtf annotation file.

The reads produced by sequencing the six RNA samples were mapped using STAR aligner [108] with default parameter settings. CountOverlap from the Bioconductor package GenomicRanges [109] was used to summarize the overlaps between the reads and the annotated features, and based on this information the Bioconductor tool edgeR [110] was employed to test for differentially expressed genes. Three pairwise comparisons were performed: (1) L- and S-morph floral RNA from *P. veris*; (2) L- and S-morph floral RNA from *P. vulgaris*; and (3) L- and S-morph leaf RNA from *P. veris*. Genes were determined to exhibit significant differential expression between samples if the *P* value (corrected for FDR) was below 0.05. Further validation of differential expression was accomplished by cross-referencing the *P. veris* L-morph versus S-morph floral genes with *P. vulgaris* L-morph versus S-morph floral genes. The set of cross-referenced genes showing significant morph-specific differential expression was examined for enrichment of gene ontology (GO) terms using the DAVID Bioinformatics Resources v6.7 [81]. The set of 113 *P. veris* genes showing morph-specific differential expression was searched against *A. thaliana* proteins (TAIR10) to identify homologous genes using blastp with an e-value cutoff of 10e-3. A total of 92 *A. thaliana* homologues were confidently identified, and the remaining 21 genes were not included in the GO terms analysis. Functional annotation clustering and enrichment analyses were performed with DAVID using default settings and the default *A. thaliana* background.

### Bulk segregant analysis to identify loci linked to the *P. veris S*-locus and PCR-based genotyping

Genomic DNA was extracted from two pools of over 100 L-morph or over 100 S-morph individuals collected from a large population of *P. veris* plants in Park Sanssouci in Potsdam, Germany, following the protocol above (see DNA Isolation, Library Preparation, and Sequencing). Restriction-associated DNA sequencing (RAD-seq; [111]) using *Pst*I was performed on these pooled genomic DNAs by Floragenex (Oregon, USA). A total of 43 and 52 million Illumina paired-end sequence reads were obtained for the L- and S-morph pools, respectively. From these paired-end sequences, contigs flanking the *Pst*I-sites were assembled, and reads from the pooled L- and S-morph samples were aligned to these contigs to detect and catalogue polymorphisms. Polymorphisms putatively linked to the *S*-locus were identified using custom perl scripts that parsed a variant call format (VCF) file cataloging the SNPs observed in the sequenced *P. veris* pools. Briefly, in order to consider a variant as linked, several criteria needed to be met. First, the sequencing coverage for any candidate allele in both pools needed to exceed 60×. The allele frequency for each variant in both the L-morph and S-morph pools was then estimated by counting the number of sequence reads in each pool harboring either the reference or alternate allele, divided by the total number of reads covering the specific variant in that pool. Note the sequencing coverage and allele counts for all loci are documented in the VCF file. Finally, in order to identify variants strongly linked to the *S*-locus we screened for variants that had an allele frequency in the L-morph pool of 1.0, while the allele frequency in S-morph pool could range between 0.3 and 0.7.

To test linkage of candidate polymorphisms to the *S*-locus, polymorphisms identified from the bulk segregant RAD-seq analysis were converted to CAPS and dCAPS markers using dCAPS Finder 2.0 [112]. Genomic DNA from individual L- and S-morph plants from the Sanssouci population was isolated using the CTAB method [94]. PCR products were digested with the appropriate restriction enzymes overnight, before resolving on 3% agarose gels. Details of the primers and corresponding restriction enzymes used are given in Additional file 1: Table S6.

### Data availability

The genome assembly and raw sequence data generated in this study are available at NCBI under the BioProject ID PRJNA238546. The data from the bulk segregant RAD-seq analysis are available under NCBI BioProject ID PRJNA268094. The Maker2 annotation files are available for download at Dryad (http://www.datadryad.org/) under doi:10.5061/dryad.2s200.

## Additional files

Additional file 1: Table S1. Summary of *de novo* genome assemblies. **Table S2.** RNAseq sequencing results for *P. veris* and *P. vulgaris.* **Table S3.** Repeat content of the *P. veris* genome assembly predicted by RepeatMasker. **Table S4.** List of genes with floral morph-specific differential expression in *P. veris* and *P. vulgaris.* **Table S5.** Functional Annotation Cluster Analysis of genes showing morph-specific differential expression. **Table S6.** Primer sequences for CAPS and dCAPS genotyping of RAD markers. Additional file 2: Figure S1. PacBio RS data. Histogram showing the distribution of **(A)** read length and **(B)** read quality from the PacBio RS data. *obconica*, *P. wilsonii*, and *P. poissonii*. Additional file 3: Figure S2. Cumulative annotation edit distance (AED). AED plotted for 5 iterative Maker2 annotations runs. Run 1 = black, run 2 = blue, run 3 = yellow, run 4 = orange, final run 5 = red. Additional file 4:
**All putatively single copy orthologous genes identified between** ***P. veris*** **,** ***P. vulgaris*** **,** ***P.***
Additional file 5:
**All putatively single copy orthologous genes identified between** ***P. veris*** **,** ***S. lycopersicum*** **(tomato),** ***M. guttatus*** **(monkeyflower),** ***V. vinifera*** **(grape), and** ***A. chinensis*** **(kiwifruit).**
Additional file 6:
**List of 24 RAD loci carrying SNPs identified as linked to the** ***P. veris S*** **-locus.**
Additional file 7: Figure S3. Comparison of *SLL1* gene between *P. vulgaris* and *P. veris*. Schematic representation of the indicated *SLL1* alleles from *P. vulgaris* and *P. veris*. Data for *P. vulgaris* are from Li *et al.* [49]. Replacement of the internal repeat by the unrelated sequence in *P. veris* was confirmed by PCR. Additional file 8: Figure S4. Test of linkage between *SLL1* haplotypes and the *S* locus in *P. veris*. **(A)** Frequency of diploid genotypes observed in L- and S-morph plants and component haplotypes. **(B)** Frequency of SLL1 haplotypes in L- and S-morph plants, and expected frequency per haplotype under the assumption of no LD between the three component SNPs. Yellow: homozygous for allele 1; blue: homozygous for allele 2; green: heterozygous. Additional file 9:
**A tab-delimited file showing the position of all SNPs based on the resequencing of a** ***P. veris*** **plant from Latvia.**
Additional file 10:
**Tab-delimited files showing the position of all SNPs identified by mapping RNAseq reads from** ***P. veris*** **L-morph flower buds, L-morph leaves, S-morph flower buds, and S-morph leaves, respectively, to the** ***P. veris*** **genome assembly.**

## Abbreviations

AED: annotation edit distance
bp: base pairs
CAPS: cleaved amplified polymorphic sequences
CDS: coding DNA sequences
CEGs: conserved eukaryotic genes
CTAB: cetrimonium bromide
dCAPS: derived cleaved amplified polymorphic sequences
FDR: false discovery rate
GO: gene ontology
indel: insertion/deletion polymorphism
LD: linkage disequilibrium
PCR: polymerase chain reaction
RAD: restriction-associated DNA
*S*-locus: self-incompatibility locus
SNP: single nucleotide polymorphism
SRA: Sequence Read Archive
UTR: untranslated region
