# Supplementary material for: The draft genome of Primula veris yields insights into the molecular basis of heterostyly
Source: Genome Biol. 2015 Jan 24;16(1):12. doi: 10.1186/s13059-014-0567-z (PMC4305239; doi:10.1186/s13059-014-0567-z)
Supplement: Additional file 1: Table S1. — Summary of de novo genome assemblies. Table S2. RNAseq sequencing results for P. veris and P. vulgaris. Table S3. Repeat content of the P. veris genome assembly predicted by RepeatMasker. Table S4. List of genes with floral morph-specific differential expression in P. veris and P. vulgaris. Table S5. Functional Annotation Cluster Analysis of genes showing morph-specific differential expression. Table S6. Primer sequences for CAPS and dCAPS genotyping of RAD markers. [file 13059_2014_567_MOESM1_ESM.doc]

**Additional file 1**

**Table S1.** Summary of *de novo* genome assemblies.

|  | **ALLPATHS** | **ALLPATHS + PBJelly** |
| --- | --- | --- |
| Contigs | 49,339 | 40,569 |
| Total contig length | 236,327,501 | 269,722,980 |
| Contig N50 | 10,076 | 13,330 |
| Longest contig | 170,541 | 233,407 |
| Scaffolds | 9,002 | 8,765 |
| Total scaffold length (including gaps) | 301,819,491 | 310,082,451 |
| Scaffold N50 | 163,948 | 165,836 |
| Largest scaffold | 2,136,047 | 2,147,216 |
| Scaffold gaps | 40,337 | 31,804 |
| Total gap length | 65,491,990 | 40,359,471 |

***Table S2.*** *RNAseq sequencing results for P. veris and P. vulgaris*.

| **RNA sample** | **Reads** |
| --- | --- |
| L-morph *P. veris* leaf | 27,595,506 |
| L-morph *P. veris* flower bud | 31,732,723 |
| S-morph *P. veris* leaf | 42,741,030 |
| S-morph *P. veris* flower bud | 45,723,409 |
| L-morph *P. vulgaris* flower bud | 31,996,681 |
| S-morph *P. vulgaris* flower bud | 29,400,331 |
| **Total** | **209,189,680** |

**Table S3.** Repeat content of the *P. veris* genome assembly predicted by RepeatMasker.

| **Repeat type predicted** | **Total number of elements** |
| --- | --- |
| SINEs | 590 |
| LINEs | 3,769 |
| LTR retroviral | 21,039 |
| DNA transposons | 16,292 |
| Low complexity | 45,964 |
| Simple repeats | 191,617 |
| Total | 279,271 |
| Total length (% of assembly) | 21.75 Mb (7%) |

**Table S4.** List of genes with floral morph-specific differential expression in *P. veris* and *P. vulgaris.* Genes in bold are those that do not exhibit differential expression between *P. veris* L- and S-morph leaf tissues. FDR represents the FDR-adjusted *P* value.

| **Gene ID** | **Scaffold** | **Strand** | ***P* value** | **FDR** | **L-morph** | **S-morph** |
| --- | --- | --- | --- | --- | --- | --- |
| **BG8816696** | Contig1404 | - | 6.59E-17 | 1.24E-13 | 0 | 568 |
| BG8816395 | Contig1741 | - | 8.61E-17 | 1.33E-13 | 374.6 | 0 |
| **BG8815255** | Contig1466 | - | 9.13E-16 | 1.29E-12 | 1,689 | 13.62 |
| **BG8815256** | Contig1466 | + | 1.22E-15 | 1.59E-12 | 1,720 | 14.48 |
| **BG8805244** | Contig478 | + | 4.13E-15 | 4.11E-12 | 252.5 | 0 |
| BG8817671 | Contig3899 | + | 5.92E-14 | 3.72E-11 | 0 | 281 |
| **BG8814694** | Contig1167 | - | 1.07E-13 | 6.49E-11 | 0 | 264 |
| **BG8817734** | Contig2067 | - | 2.04E-13 | 1.15E-10 | 30.53 | 2,152 |
| **BG8806721** | Contig98 | + | 1.28E-12 | 6.37E-10 | 286.5 | 2.555 |
| **BG8818041** | Contig2842 | - | 5.75E-12 | 2.50E-09 | 1.174 | 246.1 |
| BG8817437 | Contig2101 | - | 1.11E-11 | 4.37E-09 | 110.4 | 0 |
| **BG8808633** | Contig308 | + | 2.16E-11 | 7.77E-09 | 2.349 | 264.8 |
| **BG8814811** | Contig1963 | + | 2.80E-11 | 9.75E-09 | 42.28 | 1,719 |
| BG8812817 | Contig1357 | - | 5.61E-11 | 1.83E-08 | 1.174 | 192.4 |
| **BG8803789** | Contig150 | + | 2.26E-10 | 6.19E-08 | 532 | 15,550 |
| **BG8814319** | Contig1875 | - | 3.54E-10 | 9.22E-08 | 3.523 | 231.6 |
| BG8809571 | Contig271 | + | 6.07E-10 | 1.51E-07 | 570.7 | 19.59 |
| **BG8805963** | Contig599 | - | 8.63E-10 | 2.12E-07 | 0 | 97.93 |
| **BG8818091** | Contig2461 | + | 1.03E-09 | 2.49E-07 | 96.3 | 0.8515 |
| **BG8800775** | Contig19 | - | 2.35E-09 | 5.17E-07 | 623.6 | 14,200 |
| **BG8818258** | Contig6813 | + | 3.30E-09 | 6.73E-07 | 84.55 | 0.8515 |
| BG8814635 | Contig1250 | + | 5.31E-09 | 1.04E-06 | 337 | 13.62 |
| BG8813649 | Contig1121 | - | 7.97E-09 | 1.50E-06 | 52.85 | 0 |
| **BG8804904** | Contig1648 | + | 8.76E-09 | 1.58E-06 | 11.74 | 330.4 |
| **BG8816406** | Contig1096 | + | 9.24E-09 | 1.65E-06 | 39.93 | 882.2 |
| **BG8817314** | Contig1652 | + | 1.05E-08 | 1.82E-06 | 69.29 | 1,435 |
| **BG8814629** | Contig1148 | + | 1.09E-08 | 1.86E-06 | 177.3 | 3,500 |
| **BG8804892** | Contig1648 | + | 1.18E-08 | 1.97E-06 | 239.6 | 4,650 |
| BG8817516 | Contig1672 | - | 1.31E-08 | 2.15E-06 | 0 | 70.68 |
| **BG8801351** | Contig81 | + | 2.25E-08 | 3.49E-06 | 130.4 | 4.258 |
| BG8814308 | Contig1286 | - | 2.65E-08 | 4.02E-06 | 0 | 64.72 |
| BG8811930 | Contig417 | - | 3.00E-08 | 4.34E-06 | 41.1 | 795.3 |
| BG8817436 | Contig2101 | - | 3.28E-08 | 4.64E-06 | 44.63 | 0 |
| **BG8817209** | Contig1900 | - | 5.68E-08 | 7.29E-06 | 0 | 58.76 |
| BG8815608 | Contig2094 | + | 6.39E-08 | 8.08E-06 | 4174 | 65,930 |
| BG8807384 | Contig582 | + | 1.39E-07 | 1.57E-05 | 11.74 | 241.8 |
| **BG8809566** | Contig520 | + | 1.45E-07 | 1.63E-05 | 288.9 | 4,237 |
| BG8817601 | Contig2010 | + | 1.47E-07 | 1.63E-05 | 0 | 51.94 |
| **BG8812553** | Contig694 | - | 2.07E-07 | 2.18E-05 | 246.6 | 15.33 |
| **BG8801802** | Contig123 | - | 4.75E-07 | 4.71E-05 | 173.8 | 11.07 |
| **BG8804789** | Contig188 | - | 4.91E-07 | 4.81E-05 | 31.71 | 0 |
| **BG8802068** | Contig40 | - | 9.95E-07 | 9.11E-05 | 297.1 | 3,500 |
| **BG8809933** | Contig528 | - | 9.92E-07 | 9.11E-05 | 0 | 40.02 |
| **BG8800110** | Contig0 | - | 1.06E-06 | 9.67E-05 | 14,550 | 1,362 |
| **BG8812980** | Contig993 | - | 1.07E-06 | 9.69E-05 | 280.7 | 3,282 |
| **BG8801928** | Contig187 | + | 1.09E-06 | 9.80E-05 | 3,643 | 338.9 |
| **BG8803868** | Contig96 | - | 1.19E-06 | 0.0001046 | 28.18 | 0 |
| **BG8813894** | Contig521 | - | 1.19E-06 | 0.0001046 | 28.18 | 0 |
| **BG8818225** | Contig8477 | - | 1.34E-06 | 0.000117 | 90.43 | 5.109 |
| **BG8803549** | Contig147 | + | 3.11E-06 | 0.000245 | 24.66 | 0 |
| **BG8811999** | Contig621 | - | 3.21E-06 | 0.000251 | 7.046 | 117.5 |
| **BG8809990** | Contig402 | - | 3.25E-06 | 0.0002526 | 18.79 | 238.4 |
| **BG8802003** | Contig35 | + | 3.47E-06 | 0.0002671 | 2.349 | 65.57 |
| BG8804448 | Contig94 | + | 3.72E-06 | 0.000284 | 11.74 | 163.5 |
| BG8815393 | Contig940 | - | 3.93E-06 | 0.0002975 | 3.523 | 77.49 |
| **BG8817134** | Contig1616 | - | 4.72E-06 | 0.0003425 | 77.51 | 796.2 |
| **BG8809406** | Contig262 | - | 5.31E-06 | 0.0003716 | 1.174 | 48.54 |
| BG8816569 | Contig1297 | + | 5.41E-06 | 0.0003759 | 226.7 | 2,200 |
| **BG8810383** | Contig488 | + | 6.02E-06 | 0.000408 | 885.5 | 97.93 |
| BG8806834 | Contig283 | - | 6.24E-06 | 0.0004211 | 22.31 | 0 |
| BG8812818 | Contig1357 | - | 9.78E-06 | 0.0006236 | 29.36 | 302.3 |
| BG8806656 | Contig557 | - | 1.13E-05 | 0.0007185 | 62.24 | 583.3 |
| **BG8803310** | Contig95 | + | 1.25E-05 | 0.0007792 | 1,340 | 164.3 |
| **BG8807904** | Contig152 | + | 2.83E-05 | 0.001556 | 6,054 | 47,030 |
| **BG8809840** | Contig504 | - | 3.63E-05 | 0.001937 | 0 | 22.99 |
| **BG8813763** | Contig604 | + | 4.41E-05 | 0.002292 | 45.8 | 3.406 |
| BG8816303 | Contig877 | - | 5.97E-05 | 0.003018 | 1.174 | 34.06 |
| **BG8805461** | Contig166 | - | 6.16E-05 | 0.003096 | 17.62 | 155.8 |
| BG8808201 | Contig980 | + | 6.46E-05 | 0.00323 | 210.2 | 1,504 |
| BG8801557 | Contig24 | + | 8.47E-05 | 0.00412 | 22.31 | 181.4 |
| **BG8810980** | Contig397 | + | 9.82E-05 | 0.004636 | 2.349 | 40.87 |
| BG8800426 | Contig22 | - | 0.0001483 | 0.006545 | 0 | 17.88 |
| BG8801719 | Contig34 | + | 0.0001555 | 0.006791 | 8.22 | 77.49 |
| BG8810100 | Contig476 | + | 0.0001949 | 0.008056 | 24.66 | 176.3 |
| BG8813958 | Contig1143 | + | 0.0001963 | 0.008095 | 28.18 | 197.6 |
| **BG8811002** | Contig724 | + | 0.0002041 | 0.008397 | 4,896 | 29,510 |
| BG8802611 | Contig59 | - | 0.0002148 | 0.008794 | 95.12 | 13.62 |
| BG8800162 | Contig0 | - | 0.000221 | 0.009025 | 61.07 | 390.9 |
| **BG8814948** | Contig1341 | + | 0.0002897 | 0.01169 | 1,447 | 8,339 |
| **BG8803133** | Contig160 | - | 0.0003039 | 0.01206 | 263.1 | 1,528 |
| BG8805675 | Contig206 | - | 0.0003269 | 0.01279 | 0 | 15.33 |
| BG8815651 | Contig1248 | + | 0.0003349 | 0.01293 | 165.6 | 28.1 |
| **BG8818133** | Contig3704 | - | 0.0003357 | 0.01293 | 18.79 | 0.8515 |
| **BG8808882** | Contig523 | + | 0.0003455 | 0.01328 | 1.174 | 25.55 |
| **BG8816690** | Contig1534 | - | 0.0003704 | 0.01398 | 389.9 | 2,192 |
| **BG8804228** | Contig115 | + | 0.0003844 | 0.01448 | 1,352 | 7,499 |
| BG8814868 | Contig2325 | - | 0.0004239 | 0.01579 | 17.62 | 119.2 |
| BG8813959 | Contig1143 | + | 0.0004899 | 0.01801 | 19.96 | 129.4 |
| BG8816100 | Contig1972 | - | 0.0005761 | 0.02016 | 0 | 13.62 |
| **BG8814157** | Contig530 | + | 0.0006337 | 0.02178 | 102.2 | 17.88 |
| **BG8816999** | Contig1514 | + | 0.0006426 | 0.022 | 9.395 | 68.97 |
| BG8811500 | Contig497 | - | 0.0006832 | 0.02306 | 319.4 | 63.86 |
| **BG8802785** | Contig60 | - | 0.000711 | 0.02372 | 104.5 | 18.73 |
| **BG8804648** | Contig113 | - | 0.0007319 | 0.02423 | 16.44 | 0.8515 |
| BG8818169 | Contig3795 | + | 0.0007405 | 0.02446 | 18.79 | 115.8 |
| BG8800760 | Contig19 | - | 0.0007491 | 0.0246 | 63.42 | 10.22 |
| **BG8805917** | Contig256 | + | 0.0007755 | 0.02527 | 0 | 12.77 |
| **BG8800905** | Contig33 | - | 0.0007825 | 0.02536 | 21.14 | 1.703 |
| **BG8800245** | Contig18 | - | 0.0008182 | 0.02626 | 2.349 | 28.95 |
| **BG8804159** | Contig116 | + | 0.0008429 | 0.02691 | 418.1 | 2,094 |
| BG8807927 | Contig218 | - | 0.0009011 | 0.02844 | 29.36 | 3.406 |
| BG8814721 | Contig9 | - | 0.001019 | 0.03144 | 32.88 | 4.258 |
| BG8809518 | Contig1437 | - | 0.001118 | 0.03395 | 180.9 | 37.47 |
| **BG8815322** | Contig1445 | - | 0.001177 | 0.03537 | 1.174 | 20.44 |
| **BG8804160** | Contig116 | + | 0.00127 | 0.03762 | 1,435 | 6,727 |
| BG8818100 | Contig3485 | + | 0.001397 | 0.04082 | 109.2 | 22.14 |
| BG8815121 | Contig1589 | + | 0.001467 | 0.0422 | 1.174 | 19.59 |
| BG8802072 | Contig40 | + | 0.001483 | 0.04259 | 49.32 | 244.4 |
| **BG8812112** | Contig850 | + | 0.001566 | 0.04482 | 4,556 | 20,670 |
| **BG8802905** | Contig69 | + | 0.001589 | 0.04542 | 11.74 | 70.68 |
| **BG8803144** | Contig44 | - | 0.00161 | 0.04578 | 28.18 | 145.6 |
| BG8816941 | Contig2519 | + | 0.001819 | 0.04979 | 75.16 | 351.7 |
| **BG8802227** | Contig25 | - | 0.001824 | 0.04987 | 1,170 | 281 |

**Table S5. Functional Annotation Cluster Analysis of genes showing morph-specific differential expression.**FDR represents the FDR-adjusted *P* value.

| **Annotation cluster 1** | Enrichment score: 4.048 |  |  |  |  |  |
| --- | --- | --- | --- | --- | --- | --- |
| **Category** | **Term** | **Count** | ***P* value** | **Fold enrichment** | **Benjamini** | **FDR** |
| SP_PIR_KEYWORDS | Hydrolase | 20 | 4.22E-07 | 3.95 | 4.64E-05 | 4.77E-04 |
| SP_PIR_KEYWORDS | Secreted | 15 | 3.58E-06 | 4.62 | 1.97E-04 | 4.05E-03 |
| SP_PIR_KEYWORDS | Glycoprotein | 15 | 1.33E-05 | 4.12 | 3.67E-04 | 1.51E-02 |
| SP_PIR_KEYWORDS | Signal | 18 | 1.40E-05 | 3.42 | 3.07E-04 | 1.58E-02 |
| GOTERM_CC_FAT | GO:0005618~cell wall | 12 | 2.00E-05 | 4.92 | 1.68E-03 | 2.15E-02 |
| GOTERM_CC_FAT | GO:0030312~external encapsulating structure | 12 | 2.29E-05 | 4.85 | 9.62E-04 | 2.46E-02 |
| GOTERM_CC_FAT | GO:0005576~extracellular region | 15 | 1.95E-04 | 3.07 | 5.45E-03 | 2.09E-01 |
| UP_SEQ_FEATURE | Signal peptide | 18 | 7.16E-04 | 2.29 | 1.41E-01 | 9.03E-01 |
| UP_SEQ_FEATURE | Glycosylation site:N-linked (GlcNAc...) | 13 | 3.29E-03 | 2.51 | 2.96E-01 | 4.08E+00 |
| GOTERM_CC_FAT | GO:0012505~endomembrane system | 17 | 5.59E-01 | 1.04 | 9.95E-01 | 1.00E+02 |
|  |  |  |  |  |  |  |
| **Annotation cluster 2** | Enrichment score: 2.318 |  |  |  |  |  |
| **Category** | **Term** | **Count** | ***P* value** | **Fold enrichment** | **Benjamini** | **FDR** |
| GOTERM_CC_FAT | GO:0005618~cell wall | 12 | 2.00E-05 | 4.92 | 1.68E-03 | 2.15E-02 |
| GOTERM_CC_FAT | GO:0030312~external encapsulating structure | 12 | 2.29E-05 | 4.85 | 9.62E-04 | 2.46E-02 |
| SP_PIR_KEYWORDS | Cell wall | 5 | 2.21E-03 | 9.07 | 3.42E-02 | 2.47E+00 |
| GOTERM_BP_FAT | GO:0007047~cell wall organization | 6 | 4.20E-03 | 5.51 | 4.53E-01 | 5.43E+00 |
| GOTERM_BP_FAT | GO:0045229~external encapsulating structure organization | 6 | 5.31E-03 | 5.21 | 3.99E-01 | 6.83E+00 |
| SP_PIR_KEYWORDS | Cell wall biogenesis/degradation | 5 | 1.40E-02 | 5.34 | 1.59E-01 | 1.48E+01 |
| GOTERM_CC_FAT | GO:0009505~plant-type cell wall | 5 | 2.12E-02 | 4.64 | 3.62E-01 | 2.05E+01 |
| GOTERM_BP_FAT | GO:0009664~plant-type cell wall organization | 3 | 3.64E-02 | 9.79 | 8.30E-01 | 3.89E+01 |
| GOTERM_BP_FAT | GO:0042545~cell wall modification | 3 | 9.56E-02 | 5.66 | 9.59E-01 | 7.36E+01 |
| SP_PIR_KEYWORDS | Disulfide bond | 5 | 2.84E-01 | 1.83 | 8.56E-01 | 9.77E+01 |
|  |  |  |  |  |  |  |
| **Annotation cluster 3** | Enrichment score: 1.724 |  |  |  |  |  |
| **Category** | **Term** | **Count** | ***P* value** | **Fold enrichment** | **Benjamini** | **FDR** |
| GOTERM_BP_FAT | GO:0010038~response to metal ion | 7 | 3.48E-03 | 4.64 | 6.33E-01 | 4.53E+00 |
| GOTERM_BP_FAT | GO:0046686~response to cadmium ion | 6 | 8.37E-03 | 4.67 | 4.53E-01 | 1.06E+01 |
| GOTERM_BP_FAT | GO:0010035~response to inorganic substance | 7 | 1.73E-02 | 3.30 | 6.32E-01 | 2.06E+01 |
| SP_PIR_KEYWORDS | Magnesium | 5 | 2.30E-02 | 4.59 | 2.08E-01 | 2.31E+01 |
| GOTERM_MF_FAT | GO:0000287~magnesium ion binding | 4 | 2.06E-01 | 2.52 | 9.97E-01 | 9.37E+01 |
|  |  |  |  |  |  |  |
| **Annotation cluster 4** | Enrichment score: 1.569 |  |  |  |  |  |
| **Category** | **Term** | **Count** | ***P* value** | **Fold enrichment** | **Benjamini** | **FDR** |
| GOTERM_MF_FAT | GO:0004091~carboxylesterase activity | 8 | 1.21E-03 | 4.82 | 1.68E-01 | 1.44E+00 |
| SP_PIR_KEYWORDS | Lipid degradation | 3 | 8.66E-02 | 6.05 | 5.09E-01 | 6.41E+01 |
| GOTERM_BP_FAT | GO:0016042~lipid catabolic process | 3 | 1.87E-01 | 3.72 | 9.81E-01 | 9.36E+01 |
|  |  |  |  |  |  |  |
| **Annotation cluster 5** | Enrichment score: 0.759295862521879 |  |  |  |  |  |
| **Category** | **Term** | **Count** | ***P* value** | **Fold enrichment** | **Benjamini** | **FDR** |
| GOTERM_MF_FAT | GO:0070279~vitamin B6 binding | 3 | 1.06E-01 | 5.33 | 9.97E-01 | 7.39E+01 |
| GOTERM_MF_FAT | GO:0030170~pyridoxal phosphate binding | 3 | 1.06E-01 | 5.33 | 9.97E-01 | 7.39E+01 |
| GOTERM_MF_FAT | GO:0019842~vitamin binding | 3 | 1.83E-01 | 3.80 | 9.98E-01 | 9.11E+01 |
| GOTERM_MF_FAT | GO:0048037~cofactor binding | 4 | 4.45E-01 | 1.61 | 9.99E-01 | 9.99E+01 |
|  |  |  |  |  |  |  |
| **Annotation cluster 6** | Enrichment score: 0.742 |  |  |  |  |  |
| **Category** | **Term** | **Count** | ***P* value** | **Fold enrichment** | **Benjamini** | **FDR** |
| GOTERM_BP_FAT | GO:0009651~response to salt stress | 4 | 1.70E-01 | 2.76 | 9.88E-01 | 9.16E+01 |
| GOTERM_BP_FAT | GO:0009628~response to abiotic stimulus | 8 | 1.75E-01 | 1.70 | 9.86E-01 | 9.23E+01 |
| GOTERM_BP_FAT | GO:0006970~response to osmotic stress | 4 | 1.99E-01 | 2.55 | 9.76E-01 | 9.47E+01 |
|  |  |  |  |  |  |  |
| **Annotation cluster 7** | Enrichment score: 0.736 |  |  |  |  |  |
| **Category** | **Term** | **Count** | ***P* value** | **Fold enrichment** | **Benjamini** | **FDR** |
| GOTERM_BP_FAT | GO:0009826~unidimensional cell growth | 3 | 1.21E-01 | 4.89 | 9.76E-01 | 8.21E+01 |
| GOTERM_BP_FAT | GO:0060560~developmental growth involved in morphogenesis | 3 | 1.21E-01 | 4.89 | 9.76E-01 | 8.21E+01 |
| GOTERM_BP_FAT | GO:0048589~developmental growth | 3 | 1.53E-01 | 4.24 | 9.87E-01 | 8.90E+01 |
| GOTERM_BP_FAT | GO:0000902~cell morphogenesis | 3 | 1.91E-01 | 3.67 | 9.78E-01 | 9.40E+01 |
| GOTERM_BP_FAT | GO:0016049~cell growth | 3 | 2.00E-01 | 3.57 | 9.71E-01 | 9.48E+01 |
| GOTERM_BP_FAT | GO:0008361~regulation of cell size | 3 | 2.17E-01 | 3.38 | 9.75E-01 | 9.61E+01 |
| GOTERM_BP_FAT | GO:0032989~cellular component morphogenesis | 3 | 2.24E-01 | 3.31 | 9.74E-01 | 9.65E+01 |
| GOTERM_BP_FAT | GO:0032535~regulation of cellular component size | 3 | 2.34E-01 | 3.21 | 9.74E-01 | 9.71E+01 |
| GOTERM_BP_FAT | GO:0040007~growth | 3 | 2.45E-01 | 3.10 | 9.75E-01 | 9.76E+01 |
|  |  |  |  |  |  |  |
| **Annotation cluster 8** | Enrichment score: 0.625 |  |  |  |  |  |
| **Category** | **Term** | **Count** | ***P* value** | **Fold enrichment** | **Benjamini** | **FDR** |
| GOTERM_BP_FAT | GO:0009628~response to abiotic stimulus | 8 | 1.75E-01 | 1.70 | 9.86E-01 | 9.23E+01 |
| GOTERM_BP_FAT | GO:0009416~response to light stimulus | 4 | 2.68E-01 | 2.18 | 9.80E-01 | 9.84E+01 |
| GOTERM_BP_FAT | GO:0009314~response to radiation | 4 | 2.85E-01 | 2.11 | 9.82E-01 | 9.88E+01 |
|  |  |  |  |  |  |  |
| **Annotation cluster 9** | Enrichment score: 0.609 |  |  |  |  |  |
| **Category** | **Term** | **Count** | ***P* value** | **Fold enrichment** | **Benjamini** | **FDR** |
| SP_PIR_KEYWORDS | Monooxygenase | 4 | 5.65E-02 | 4.56 | 3.88E-01 | 4.81E+01 |
| GOTERM_MF_FAT | GO:0019825~oxygen binding | 4 | 9.39E-02 | 3.65 | 9.99E-01 | 6.93E+01 |
| SP_PIR_KEYWORDS | Heme | 4 | 1.12E-01 | 3.38 | 5.60E-01 | 7.40E+01 |
| GOTERM_MF_FAT | GO:0046906~tetrapyrrole binding | 5 | 1.18E-01 | 2.63 | 9.91E-01 | 7.77E+01 |
| COG_ONTOLOGY | Secondary metabolites biosynthesis, transport, and catabolism | 4 | 1.29E-01 | 2.88 | 6.68E-01 | 5.30E+01 |
| SP_PIR_KEYWORDS | Iron | 5 | 1.71E-01 | 2.29 | 7.04E-01 | 8.80E+01 |
| UP_SEQ_FEATURE | Metal ion-binding site:Iron (heme axial ligand) | 3 | 2.18E-01 | 3.35 | 1.00E+00 | 9.56E+01 |
| GOTERM_MF_FAT | GO:0020037~heme binding | 4 | 2.51E-01 | 2.27 | 9.98E-01 | 9.68E+01 |
| SP_PIR_KEYWORDS | Oxidoreductase | 5 | 3.97E-01 | 1.55 | 9.30E-01 | 9.97E+01 |
| GOTERM_MF_FAT | GO:0005506~iron ion binding | 5 | 4.39E-01 | 1.46 | 9.99E-01 | 9.99E+01 |
| GOTERM_BP_FAT | GO:0055114~oxidation reduction | 6 | 4.87E-01 | 1.29 | 9.99E-01 | 1.00E+02 |
| GOTERM_MF_FAT | GO:0009055~electron carrier activity | 4 | 5.95E-01 | 1.29 | 1.00E+00 | 1.00E+02 |
| SP_PIR_KEYWORDS | Metal-binding | 7 | 6.90E-01 | 1.01 | 9.82E-01 | 1.00E+02 |
| GOTERM_MF_FAT | GO:0046914~transition metal ion binding | 6 | 9.98E-01 | 0.46 | 1.00E+00 | 1.00E+02 |
|  |  |  |  |  |  |  |
| **Annotation cluster 10** | Enrichment score: 0.464 |  |  |  |  |  |
| **Category** | **Term** | **Count** | ***P* value** | **Fold enrichment** | **Benjamini** | **FDR** |
| SP_PIR_KEYWORDS | Membrane | 13 | 1.03E-01 | 1.60 | 5.50E-01 | 7.08E+01 |
| GOTERM_CC_FAT | GO:0031224~intrinsic to membrane | 14 | 2.31E-01 | 1.32 | 9.36E-01 | 9.40E+01 |
| SP_PIR_KEYWORDS | Transmembrane | 10 | 2.34E-01 | 1.46 | 8.05E-01 | 9.51E+01 |
| UP_SEQ_FEATURE | Transmembrane region | 10 | 4.93E-01 | 1.14 | 1.00E+00 | 1.00E+02 |
| SP_PIR_KEYWORDS | Transport | 5 | 5.14E-01 | 1.33 | 9.58E-01 | 1.00E+02 |
| UP_SEQ_FEATURE | Topological domain:Cytoplasmic | 4 | 6.24E-01 | 1.23 | 1.00E+00 | 1.00E+02 |
| GOTERM_CC_FAT | GO:0016021~integral to membrane | 9 | 6.45E-01 | 1.03 | 9.97E-01 | 1.00E+02 |
|  |  |  |  |  |  |  |
| **Annotation cluster 11** | Enrichment score: 0.392 |  |  |  |  |  |
| **Category** | **Term** | **Count** | ***P* value** | **Fold enrichment** | **Benjamini** | **FDR** |
| GOTERM_BP_FAT | GO:0010033~response to organic substance | 7 | 2.99E-01 | 1.51 | 9.80E-01 | 9.91E+01 |
| GOTERM_BP_FAT | GO:0009719~response to endogenous stimulus | 6 | 3.23E-01 | 1.57 | 9.84E-01 | 9.94E+01 |
| GOTERM_BP_FAT | GO:0009725~response to hormone stimulus | 5 | 4.69E-01 | 1.40 | 9.98E-01 | 1.00E+02 |
| GOTERM_BP_FAT | GO:0007242~intracellular signaling cascade | 4 | 5.95E-01 | 1.29 | 1.00E+00 | 1.00E+02 |
|  |  |  |  |  |  |  |
| **Annotation cluster 12** | Enrichment score: 0.383 |  |  |  |  |  |
| **Category** | **Term** | **Count** | ***P* value** | **Fold enrichment** | **Benjamini** | **FDR** |
| GOTERM_MF_FAT | GO:0043169~cation binding | 20 | 2.91E-01 | 1.19 | 9.99E-01 | 9.84E+01 |
| GOTERM_MF_FAT | GO:0043167~ion binding | 20 | 2.95E-01 | 1.18 | 9.97E-01 | 9.85E+01 |
| GOTERM_MF_FAT | GO:0046872~metal ion binding | 14 | 8.28E-01 | 0.87 | 1.00E+00 | 1.00E+02 |
|  |  |  |  |  |  |  |
| **Annotation cluster 13** | Enrichment score: 0.142 |  |  |  |  |  |
| **Category** | **Term** | **Count** | ***P* value** | **Fold enrichment** | **Benjamini** | **FDR** |
| SP_PIR_KEYWORDS | Nucleus | 9 | 4.07E-01 | 1.27 | 9.27E-01 | 9.97E+01 |
| SP_PIR_KEYWORDS | DNA-binding | 6 | 5.27E-01 | 1.24 | 9.58E-01 | 1.00E+02 |
| SP_PIR_KEYWORDS | Transcription regulation | 5 | 5.83E-01 | 1.22 | 9.72E-01 | 1.00E+02 |
| SP_PIR_KEYWORDS | Transcription | 5 | 6.00E-01 | 1.19 | 9.73E-01 | 1.00E+02 |
| GOTERM_MF_FAT | GO:0043565~sequence-specific DNA binding | 3 | 6.63E-01 | 1.31 | 1.00E+00 | 1.00E+02 |
| GOTERM_BP_FAT | GO:0006350~transcription | 5 | 7.57E-01 | 0.97 | 1.00E+00 | 1.00E+02 |
| GOTERM_BP_FAT | GO:0006355~regulation of transcription, DNA-dependent | 4 | 8.02E-01 | 0.93 | 1.00E+00 | 1.00E+02 |
| GOTERM_BP_FAT | GO:0051252~regulation of RNA metabolic process | 4 | 8.05E-01 | 0.93 | 1.00E+00 | 1.00E+02 |
| GOTERM_BP_FAT | GO:0045449~regulation of transcription | 6 | 9.07E-01 | 0.75 | 1.00E+00 | 1.00E+02 |
| GOTERM_MF_FAT | GO:0003677~DNA binding | 8 | 9.51E-01 | 0.69 | 1.00E+00 | 1.00E+02 |
| GOTERM_MF_FAT | GO:0003700~transcription factor activity | 5 | 9.61E-01 | 0.63 | 1.00E+00 | 1.00E+02 |
| GOTERM_MF_FAT | GO:0030528~transcription regulator activity | 5 | 9.83E-01 | 0.55 | 1.00E+00 | 1.00E+02 |
|  |  |  |  |  |  |  |
| **Annotation cluster 14** | Enrichment score: 0.130 |  |  |  |  |  |
| **Category** | **Term** | **Count** | ***P* value** | **Fold enrichment** | **Benjamini** | **FDR** |
| SP_PIR_KEYWORDS | Transit peptide | 4 | 6.57E-01 | 1.18 | 9.80E-01 | 1.00E+02 |
| SP_PIR_KEYWORDS | Plastid | 3 | 7.35E-01 | 1.15 | 9.85E-01 | 1.00E+02 |
| SP_PIR_KEYWORDS | Chloroplast | 3 | 7.52E-01 | 1.11 | 9.86E-01 | 1.00E+02 |
| UP_SEQ_FEATURE | Transit peptide:Chloroplast | 3 | 8.35E-01 | 0.93 | 1.00E+00 | 1.00E+02 |
|  |  |  |  |  |  |  |
| **Annotation cluster 15** | Enrichment score: 0.127 |  |  |  |  |  |
| **Category** | **Term** | **Count** | ***P* value** | **Fold enrichment** | **Benjamini** | **FDR** |
| GOTERM_BP_FAT | GO:0009791~post-embryonic development | 4 | 6.46E-01 | 1.20 | 1.00E+00 | 1.00E+02 |
| GOTERM_BP_FAT | GO:0048608~reproductive structure development | 3 | 7.81E-01 | 1.04 | 1.00E+00 | 1.00E+02 |
| GOTERM_BP_FAT | GO:0003006~reproductive developmental process | 3 | 8.26E-01 | 0.95 | 1.00E+00 | 1.00E+02 |
|  |  |  |  |  |  |  |
| **Annotation cluster 16** | Enrichment score: 0.109 |  |  |  |  |  |
| **Category** | **Term** | **Count** | ***P* value** | **Fold enrichment** | **Benjamini** | **FDR** |
| GOTERM_CC_FAT | GO:0009579~thylakoid | 3 | 5.78E-01 | 1.53 | 9.94E-01 | 1.00E+02 |
| GOTERM_CC_FAT | GO:0044434~chloroplast part | 3 | 8.96E-01 | 0.79 | 1.00E+00 | 1.00E+02 |
| GOTERM_CC_FAT | GO:0044435~plastid part | 3 | 9.06E-01 | 0.76 | 1.00E+00 | 1.00E+02 |
|  |  |  |  |  |  |  |
| **Annotation Cluster 17** | Enrichment Score: 0.068 |  |  |  |  |  |
| **Category** | **Term** | **Count** | ***P* value** | **Fold enrichment** | **Benjamini** | **FDR** |
| UP_SEQ_FEATURE | Nucleotide phosphate-binding region:ATP | 5 | 5.59E-01 | 1.25 | 1.00E+00 | 1.00E+02 |
| SP_PIR_KEYWORDS | Kinase | 4 | 6.51E-01 | 1.19 | 9.82E-01 | 1.00E+02 |
| SP_PIR_KEYWORDS | Nucleotide-binding | 7 | 6.59E-01 | 1.04 | 9.78E-01 | 1.00E+02 |
| SP_PIR_KEYWORDS | Serine/threonine-protein kinase | 3 | 6.97E-01 | 1.23 | 9.81E-01 | 1.00E+02 |
| SP_PIR_KEYWORDS | ATP-binding | 6 | 7.08E-01 | 1.01 | 9.81E-01 | 1.00E+02 |
| GOTERM_BP_FAT | GO:0006796~phosphate metabolic process | 4 | 8.80E-01 | 0.80 | 1.00E+00 | 1.00E+02 |
| GOTERM_BP_FAT | GO:0006793~phosphorus metabolic process | 4 | 8.80E-01 | 0.80 | 1.00E+00 | 1.00E+02 |
| GOTERM_MF_FAT | GO:0001883~purine nucleoside binding | 9 | 9.21E-01 | 0.75 | 1.00E+00 | 1.00E+02 |
| GOTERM_MF_FAT | GO:0030554~adenyl nucleotide binding | 9 | 9.21E-01 | 0.75 | 1.00E+00 | 1.00E+02 |
| GOTERM_MF_FAT | GO:0017076~purine nucleotide binding | 10 | 9.22E-01 | 0.76 | 1.00E+00 | 1.00E+02 |
| GOTERM_MF_FAT | GO:0001882~nucleoside binding | 9 | 9.23E-01 | 0.75 | 1.00E+00 | 1.00E+02 |
| GOTERM_BP_FAT | GO:0006468~protein amino acid phosphorylation | 3 | 9.24E-01 | 0.72 | 1.00E+00 | 1.00E+02 |
| GOTERM_MF_FAT | GO:0032559~adenyl ribonucleotide binding | 8 | 9.40E-01 | 0.71 | 1.00E+00 | 1.00E+02 |
| GOTERM_MF_FAT | GO:0004674~protein serine/threonine kinase activity | 3 | 9.47E-01 | 0.65 | 1.00E+00 | 1.00E+02 |
| GOTERM_BP_FAT | GO:0016310~phosphorylation | 3 | 9.49E-01 | 0.65 | 1.00E+00 | 1.00E+02 |
| GOTERM_MF_FAT | GO:0000166~nucleotide binding | 11 | 9.65E-01 | 0.70 | 1.00E+00 | 1.00E+02 |
| GOTERM_MF_FAT | GO:0004672~protein kinase activity | 3 | 9.70E-01 | 0.57 | 1.00E+00 | 1.00E+02 |
| GOTERM_MF_FAT | GO:0005524~ATP binding | 7 | 9.71E-01 | 0.63 | 1.00E+00 | 1.00E+02 |
| GOTERM_MF_FAT | GO:0032553~ribonucleotide binding | 8 | 9.72E-01 | 0.65 | 1.00E+00 | 1.00E+02 |
| GOTERM_MF_FAT | GO:0032555~purine ribonucleotide binding | 8 | 9.72E-01 | 0.65 | 1.00E+00 | 1.00E+02 |

**Table S6. Primer sequences for CAPS and dCAPS genotyping of RAD markers.**

| **Marker** | **Primer sequences** | **Annealing temperature (°C)** | **Digestion enzyme** | **Size of fragments for L-morph allele (bp)** | **Size of fragments for S-morph allele (bp)** |
| --- | --- | --- | --- | --- | --- |
| 9274 | AATCCCACCAACCCAATGTA | 58 | Cac8I | 167 | 147, 20 |
|  | TAACAGTCTTCTAGCAAGTTCGCTT |  |  |  |  |
| 22125 | GCTTTGAGATTGTGAAATACTGGCT | 58 | NheI | 136, 25 | 161 |
|  | CCAATCATGTGCCTGTTTTC |  |  |  |  |
| 23038 | TGCAGAACCAGAGTTTGTCACT | 58 | BcgI | 147, 17 | 164 |
|  | AAGACATCAGTGTCACGACATATTT |  |  |  |  |
| 37812 | CAAAACCCATTACCCCGAAC | 58 | RsaI | 188, 21 | 209 |
|  | TAACCTTATTAGCTAATGTTGTGTA |  |  |  |  |
| 40019 | TTCTTCCTGATAATCTTAATATCCG | 58 | AciI | 174, 15 | 199 |
|  | CAAACAATCAGCAATCAGGG |  |  |  |  |
| 41358 | CATGCCTGCATGTGTCACTTA | 58 | ApaLI | 105, 55 | 160 |
|  | AGCTTTCAGCGTTTGTGACC |  |  |  |  |
| 44394 | GGGAATAAATCACAGCAATGAA | 58 | Cac8I | 95, 25 | 120 |
|  | TGCTGCATTTTTTATCCTTGGCTAG |  |  |  |  |
| 51955 | AAAGTTTCGGGCCAAAGAAT | 58 | RsaI | 107, 83 | 190 |
|  | GTAGTGGCGGACTTTGTTCC |  |  |  |  |
| 52968 | GCAAAGCCGATATCATAGCC | 58 | HgaI | 129, 101 | 230 |
|  | AGCATTAGCAAAAGCCCAAC |  |  |  |  |
| 55364 | TAATCTTTGTTAGCTTTCAGAACAT | 58 | NspI | 160 | 135, 25 |
|  | CAGCGAAACAGGTTTTTCTTATT |  |  |  |  |
| 57656 | TGATAACAAAGGTGTTAGAGACAGC | 56 | BbvI | 230 | 196, 34 |
|  | TGCAATGAGAAGGAGGGGTA |  |  |  |  |
| 59102 | AATTTCTGGGATCTTGTGTGTGTAC | 58 | BsaAI | 124 | 99, 25 |
|  | GCGGTAATAAAATTCGGCAAT |  |  |  |  |
| 60550 | TCGGAGATTCCTGTTGAGAGA | 58 | Cac8I | 135, 30 | 165 |
|  | TAAACCAAACAACATATAAAGCATG |  |  |  |  |
| 62912 | TGAACAATCCCCTCAGAAGC | 58 | BbvI | 153, 136 | 289 |
|  | TGAGGGTTATTGCTCCCTTG |  |  |  |  |
| 66794 | AGCATTTTGAAGAAATTTTAAATCC | 58 | AciL | 147, 24 | 171 |
|  | TGACATCTGCCACAGTAACCTC |  |  |  |  |
| 72585 | CATTGCTCGAGGGATTCAGT | 58 | BcgI | 166, 25 | 191 |
|  | TTTATGAAAGGAACCCGAAAAAGGT |  |  |  |  |
| 73865 | ATAGGGCACGGCTAAACAAA | 58 | AccI | 244, 24 | 268 |
|  | AAACCATACTACCCTTAAAGAGTAT | |  |  |  |
| 79269 | TATTACGCGTCTCACACGCAGCGCG | 58 | Cac8I | 148 | 125, 23 |
|  | TGCAGCAATTTGGTGAAGAT |  |  |  |  |
| 83053 | ATGCTCTCATATGGGAACATAACTA | 58 | MslI | 297 | 269, 28 |
|  | TGCATTGAGTGTATGTGCATGT |  |  |  |  |
| 83452 | GAGGATTCAGGGTGCAAAGA | 58 | NlaIII | 131 | 105, 26 |
|  | GTGGAAGCAAGTCTACTCCTACCAT |  |  |  |  |
| 93610 | TTAATATGCTTCAGTTAAGGTGATC | 58 | BclI | 146, 22 | 168 |
|  | TGCAGGTAATTGTGCCATGT |  |  |  |  |
| 99054 | TAGCCCCTTGTGATTGAAGG | 58 | NdeI | 139, 24 | 163 |
|  | AAGCTACAACCCATTTTTGTACATA |  |  |  |  |
| 100779 | CGAGTATACCACGCTCACCA | 58 | HpaI | 161, 23 | 184 |
|  | ATATTTCGGCCATGTTCGTTGTTAA |  |  |  |  |
| 101982 | TCGCCATCACCTCATCATTA | 58 | NdeI | 234 | 210, 24 |
|  | TTCACGAGCCACCATACTTCAAGCA |  |  |  |  |
