# Supplementary figures and images for: The draft genome of Primula veris yields insights into the molecular basis of heterostyly
Source: Genome Biol. 2015 Jan 24;16(1):12. doi: 10.1186/s13059-014-0567-z (PMC4305239; doi:10.1186/s13059-014-0567-z)

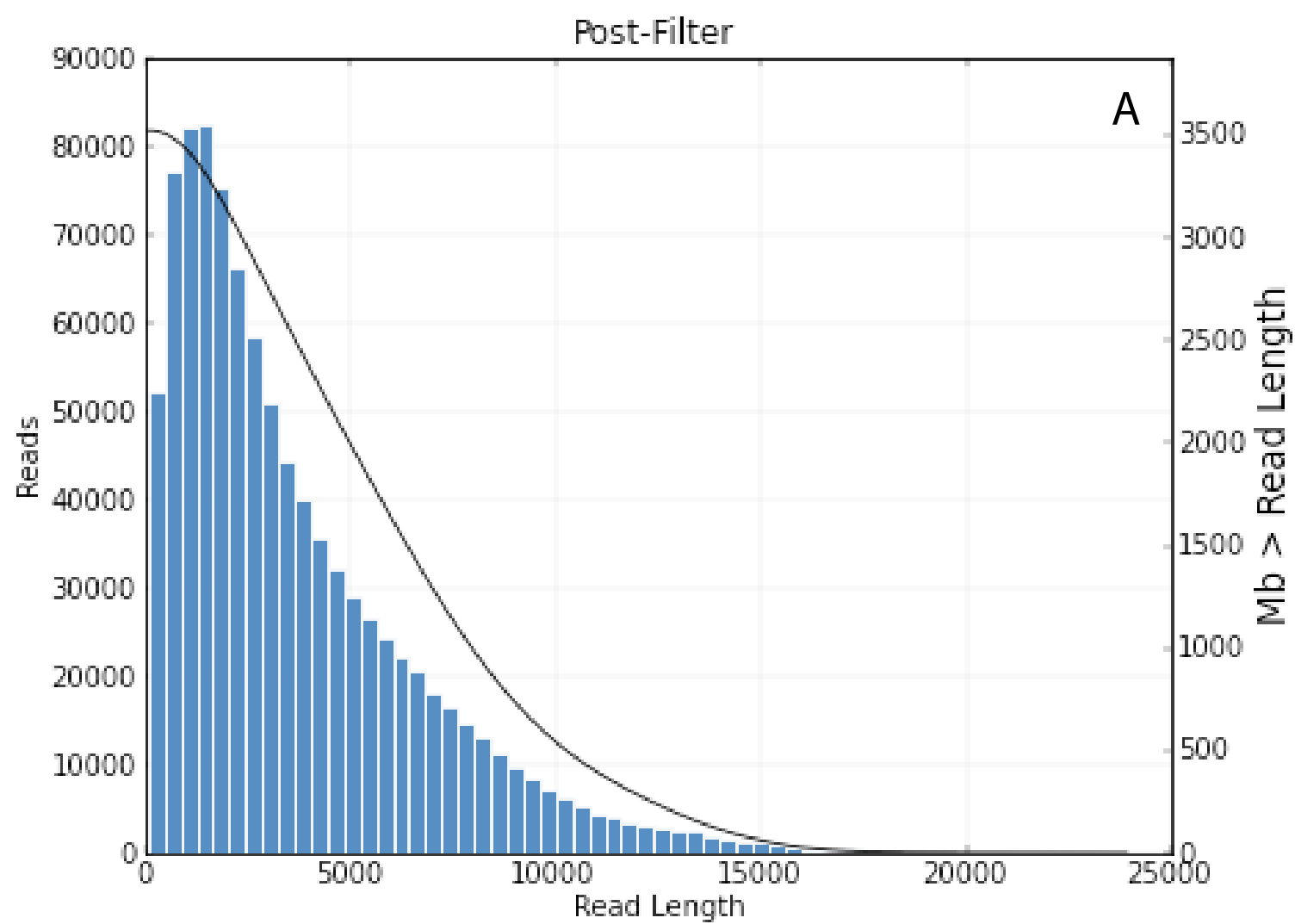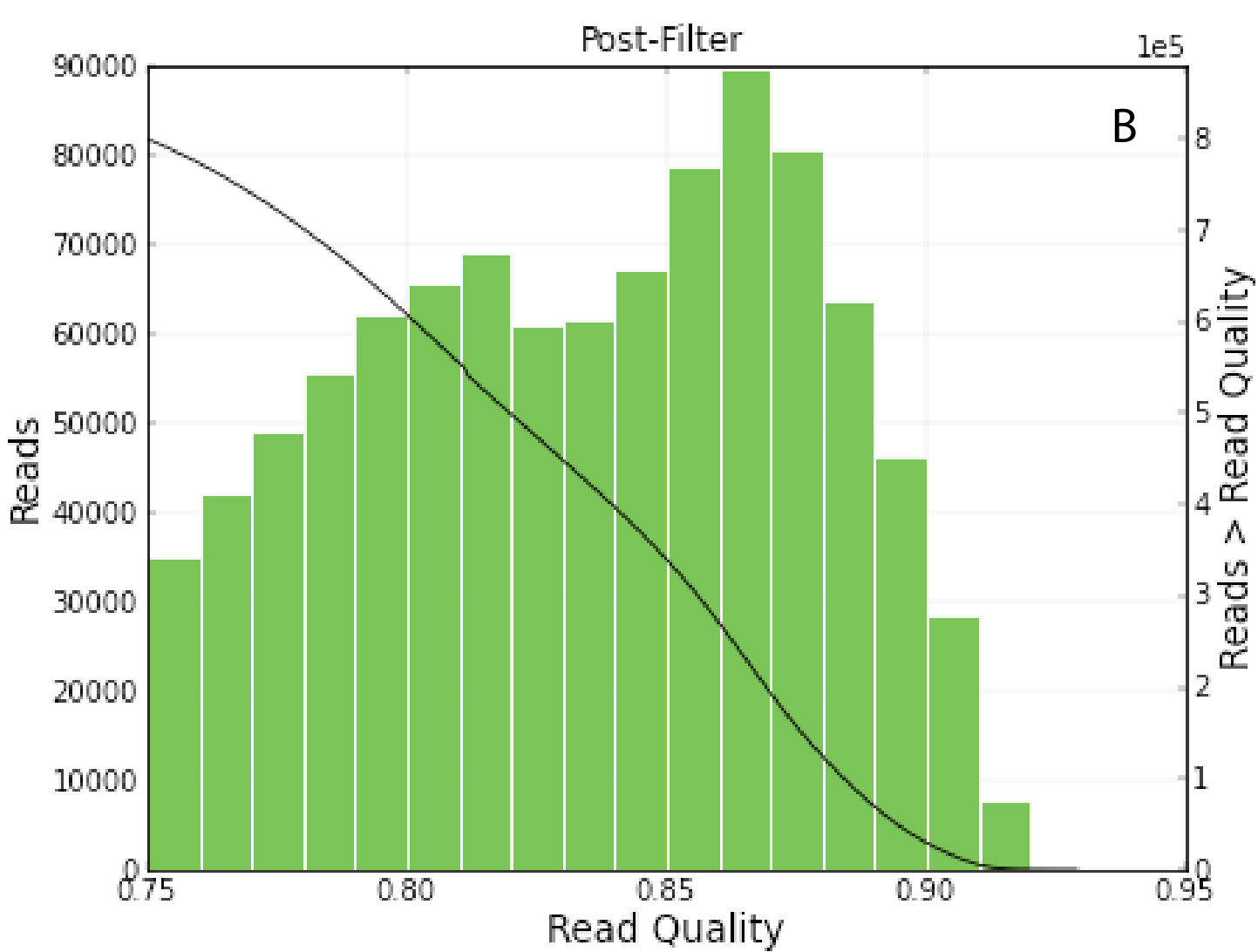

Supplement: Additional file 2: Figure S1. — PacBio RS data. Histogram showing the distribution of (A) read length and (B) read quality from the PacBio RS data. obconica, P. wilsonii, and P. poissonii. [file 13059_2014_567_MOESM2_ESM.pdf]

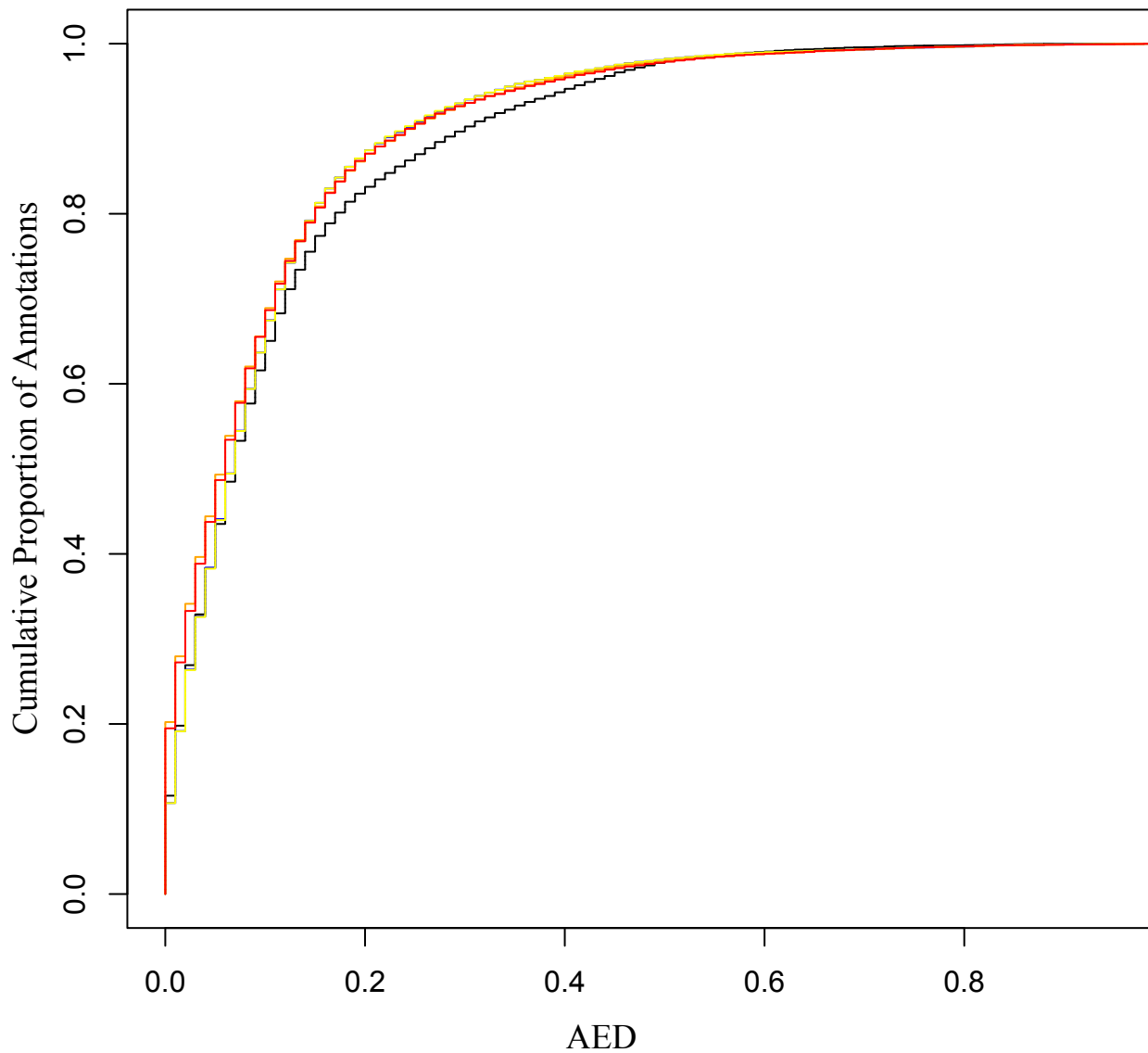

Supplement: Additional file 3: Figure S2. — Cumulative annotation edit distance (AED). AED plotted for 5 iterative Maker2 annotations runs. Run 1 = black, run 2 = blue, run 3 = yellow, run 4 = orange, final run 5 = red. [file 13059_2014_567_MOESM3_ESM.pdf]

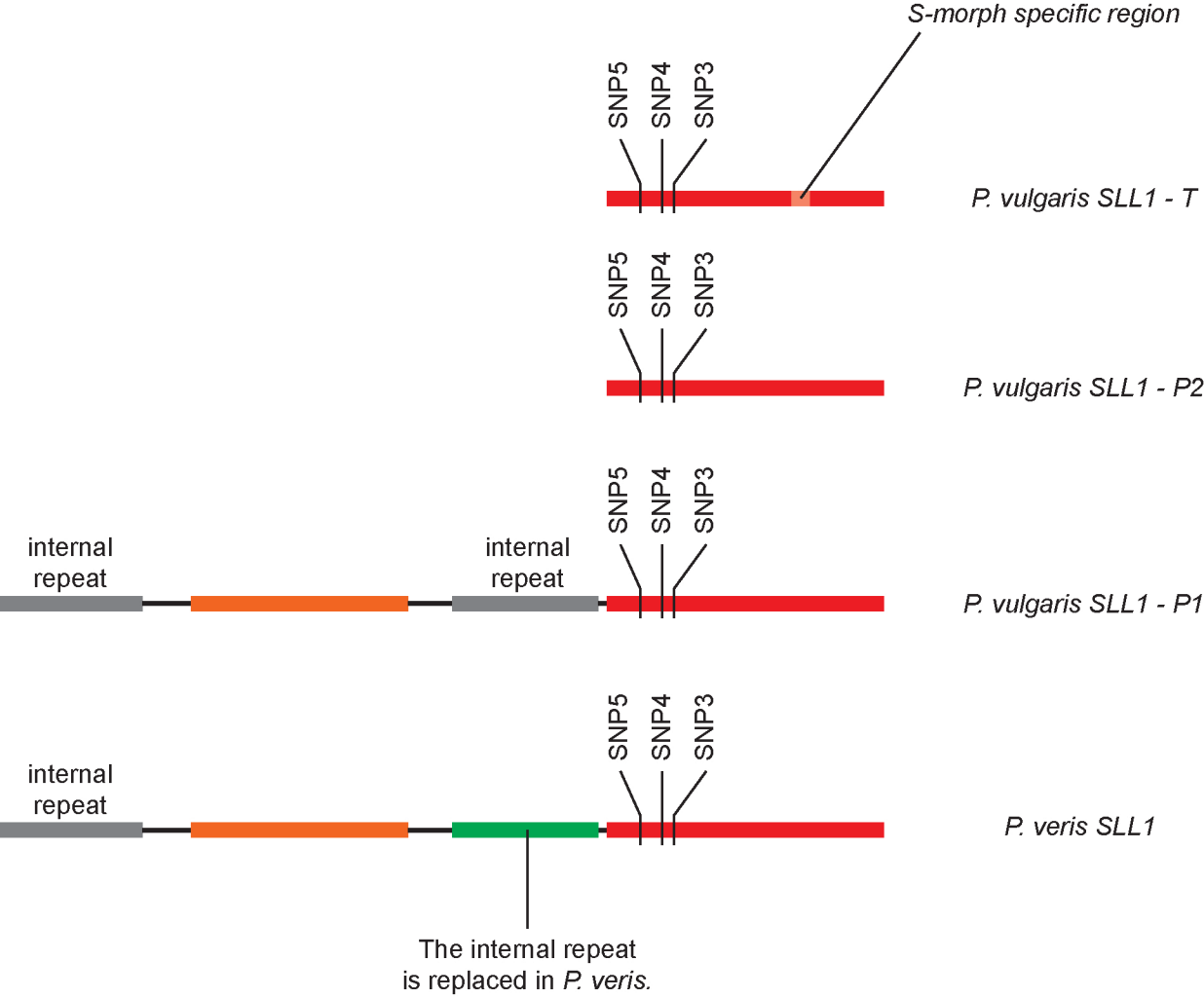

Supplement: Additional file 7: Figure S3. — Comparison of SLL1 gene between P. vulgaris and P. veris. Schematic representation of the indicated SLL1 alleles from P. vulgaris and P. veris. Data for P. vulgaris are from Li et al. [49]. Replacement of the internal repeat by the unrelated sequence in P. veris was confirmed by PCR. [file 13059_2014_567_MOESM7_ESM.pdf]

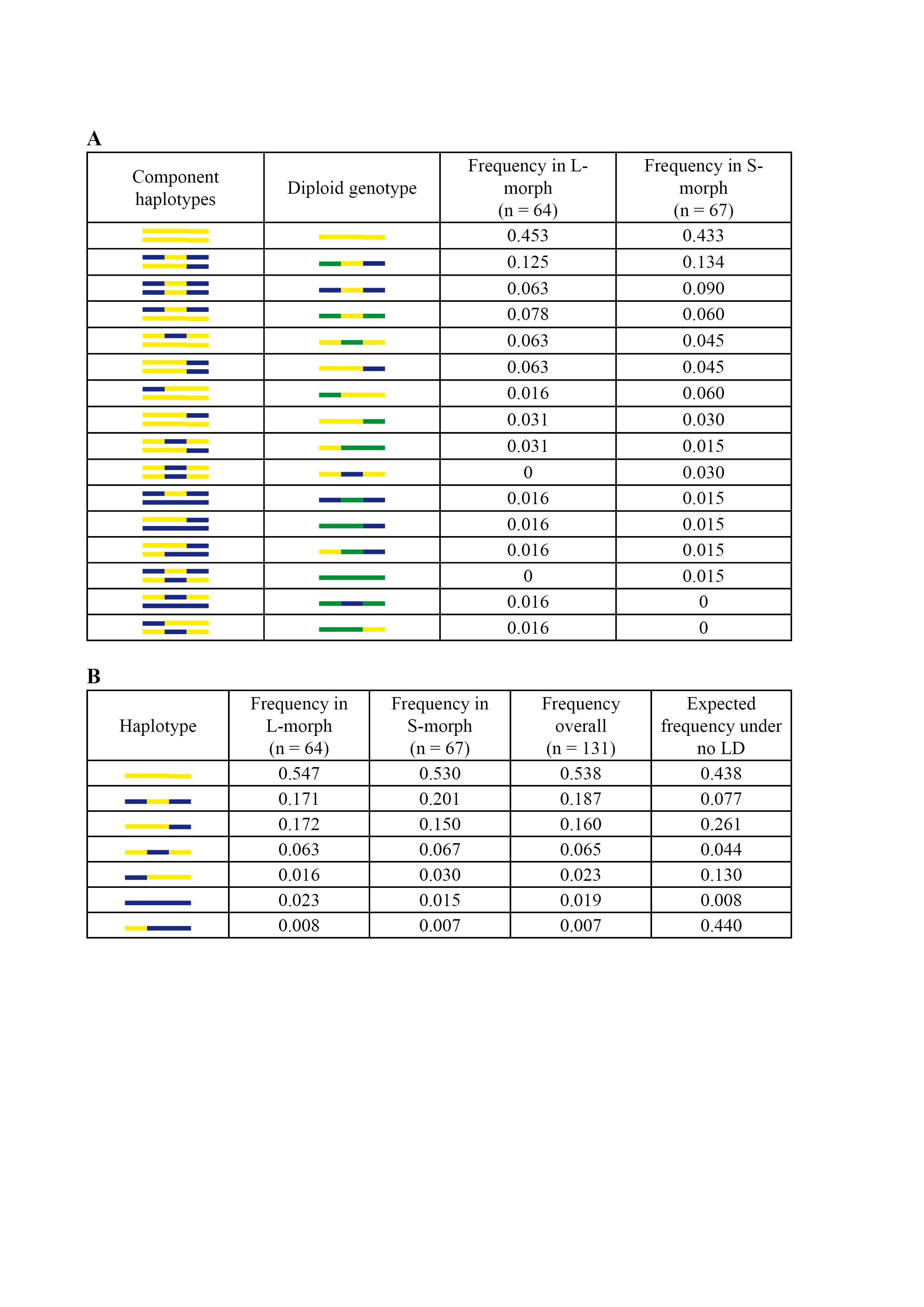

Supplement: Additional file 8: Figure S4. — Test of linkage between SLL1 haplotypes and the S locus in P. veris. (A) Frequency of diploid genotypes observed in L- and S-morph plants and component haplotypes. (B) Frequency of SLL1 haplotypes in L- and S-morph plants, and expected frequency per haplotype under the assumption of no LD between the three component SNPs. Yellow: homozygous for allele 1; blue: homozygous for allele 2; green: heterozygous. [file 13059_2014_567_MOESM8_ESM.jpeg]
